# Ruthenium Polypyridyl Complex Bound to a Unimolecular Chair-Form G-Quadruplex

**DOI:** 10.1021/jacs.2c00178

**Published:** 2022-03-24

**Authors:** Kane T. McQuaid, Shuntaro Takahashi, Lena Baumgaertner, David J. Cardin, Neil G. Paterson, James P. Hall, Naoki Sugimoto, Christine J. Cardin

**Affiliations:** † Department of Chemistry, University of Reading, Whiteknights, Reading RG6 6AD, U.K.; ‡ FIBER (Frontier Institute for Biomolecular Engineering Research), Konan University, 7-1-20 Minatojima-minamimachi, Chuo-ku, Kobe 650-0047, Japan; § Diamond Light Source Ltd., Harwell Science and Innovation Campus, Didcot, Oxfordshire OX11 0DE, U.K.; ∥ School of Pharmacy, University of Reading, Whiteknights, Reading RG6 6AD, U.K.; ⊥ FIRST (Graduate School of Frontiers of Innovative Research in Science and Technology), Konan University, 7-1-20 Minatojima-Minamimashi, Chuo-Ku, Kobe 650-0047, Japan

## Abstract

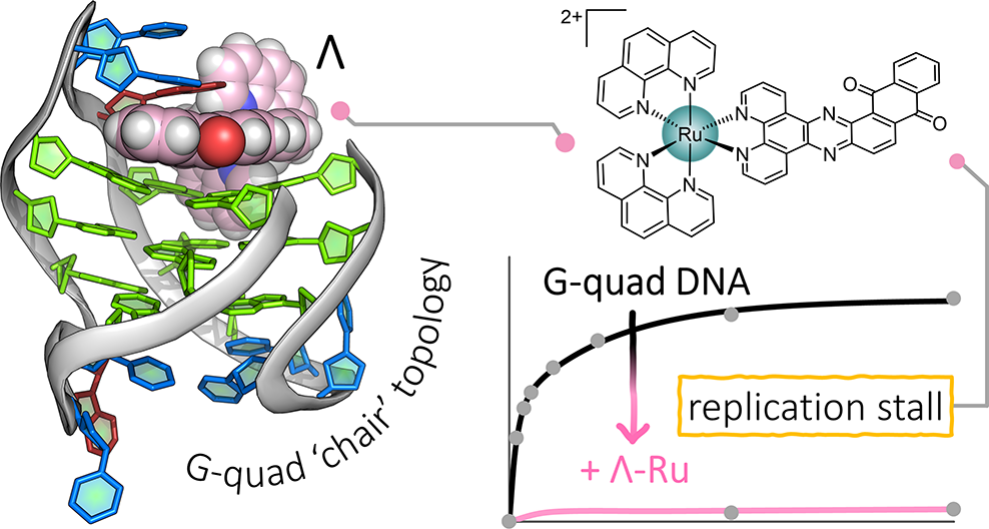

The DNA G-quadruplex is known for forming a range of topologies and for the observed lability of the assembly, consistent with its transient formation in live cells. The stabilization of a particular topology by a small molecule is of great importance for therapeutic applications. Here, we show that the ruthenium complex Λ-[Ru(phen)_2_(qdppz)]^2+^ displays enantiospecific G-quadruplex binding. It crystallized in 1:1 stoichiometry with a modified human telomeric G-quadruplex sequence, GGGTTAGGGTTAGGGTTTGGG (*htel21*T_18_), in an antiparallel chair topology, the first structurally characterized example of ligand binding to this topology. The lambda complex is bound in an intercalation cavity created by a terminal G-quartet and the central narrow lateral loop formed by T_10_–T_11_–A_12_. The two remaining wide lateral loops are linked through a third K^+^ ion at the other end of the G-quartet stack, which also coordinates three thymine residues. In a comparative ligand-binding study, we showed, using a Klenow fragment assay, that this complex is the strongest observed inhibitor of replication, both using the native human telomeric sequence and the modified sequence used in this work.

## Introduction

The extreme flexibility of a single DNA strand is in marked contrast to the limited range of structures known for the familiar double helical DNA.^1^ The flexibility includes both that of the DNA sugar–phosphate backbone and that of the orientation of the DNA bases relative to the sugar rings. Single-stranded DNA is found naturally at the ends of chromosomes, which in humans consists of several hundred repeats of the telomeric base sequence (T_2_AG_3_)_*n*_.^2−5^ In this case, a specific structure composed of guanosine residues, the G-quadruplex or G4, can form in the presence of K^+^ ions, which are naturally present at ∼100 mM concentrations in human cells. The resulting assembly is known from X-ray^6^ and nuclear magnetic resonance (NMR)^4^ studies to give a range of folds of the DNA backbone, held together by the central K^+^ ions and made possible by the rotation of the guanine base relative to its attached sugar. These studies have been carried out on variants of the G_3_(T_2_AG_3_)_3_ minimal sequence, the shortest sequence giving a unimolecular quadruplex based on the human telomeric sequence. Furthermore, G4 motifs are often found in the promoter regions of cancer-related genes, with expression suppressed and with loss of the G4 structure often causing gene activation.^7^ Therefore, G4-targeting compounds have been widely studied,^8^ for example, for imaging the activity of promoter regions, suppressing activation via stabilization of the motifs or for targeted site-specific damage. A major goal is that of specific G4 recognition, which could have therapeutic value,^9−11^ as recently demonstrated by the NMR study of epiberberine stabilizing the hybrid-2 form of the human telomeric sequence.^12,13^ Noteworthy, though, is the small number of such completed structural studies, because to carry out such studies, whether by NMR or X-ray crystallography, a single topology has to be generated and stabilized.^14^ For that to be possible, each guanosine nucleoside in the G4 has to adopt a preferred orientation (*syn*/*anti*) about the base–sugar bond. For example, if all the orientations are *anti*, a parallel stranded G4 is formed, with the −GGG– triplets connected by propeller loops.^15^ In this study, we demonstrate the use of a ruthenium polypyridyl complex for this purpose, driven by the preference to interact with a guanosine residue having a *syn* conformation.^16,17^

Ruthenium polypyridyl complexes are of interest for their application as cellular probes and anticancer therapeutic agents.^18−22^ Their photophysical properties and modular design allow wide tunability of the ligands to target specific DNA and RNA topologies, including those associated with G4s.^23,24^ Using X-ray crystallography, we studied these complexes as DNA-binding ligands, and those studies have recently been extended to G4 binding.^17,25,26^ The parent complex [Ru(phen)_2_(dppz)]^2+^ and its photooxidizing analogue [Ru(TAP)_2_(dppz)]^2+^ bind relatively nonspecifically to DNA, and have been crystallized with a range of duplex-forming sequences.^27−29^

We now report the first X-ray crystallographic study of any ligand with a unimolecular G-quadruplex showing a non-parallel DNA topology in the crystal. In 2019, the first structural report of this antiparallel chair topology followed from a study of several naturally occurring variants of the human telomeric sequence (T_2_AG_3_)_*n*_.^30^ Here, we report that an antiparallel chair topology is stabilized by Λ-[Ru(phen)_2_(qdppz)]^2+^ (Λ-(**I**)), using the same sequence, *htel21*T_18_. We used the modified sequence reported in this paper after years of using native telomeric sequences in crystallization trials without success. Using the same ruthenium complex, we experienced mainly precipitation when mixed with the native *htel21* sequence, which we ascribed to the known heterogeneity of this sequence in a solution.^4^ Crystallization cannot normally succeed unless a single assembly is formed in the crystallization mixture.

## Results

**Figure 1:**
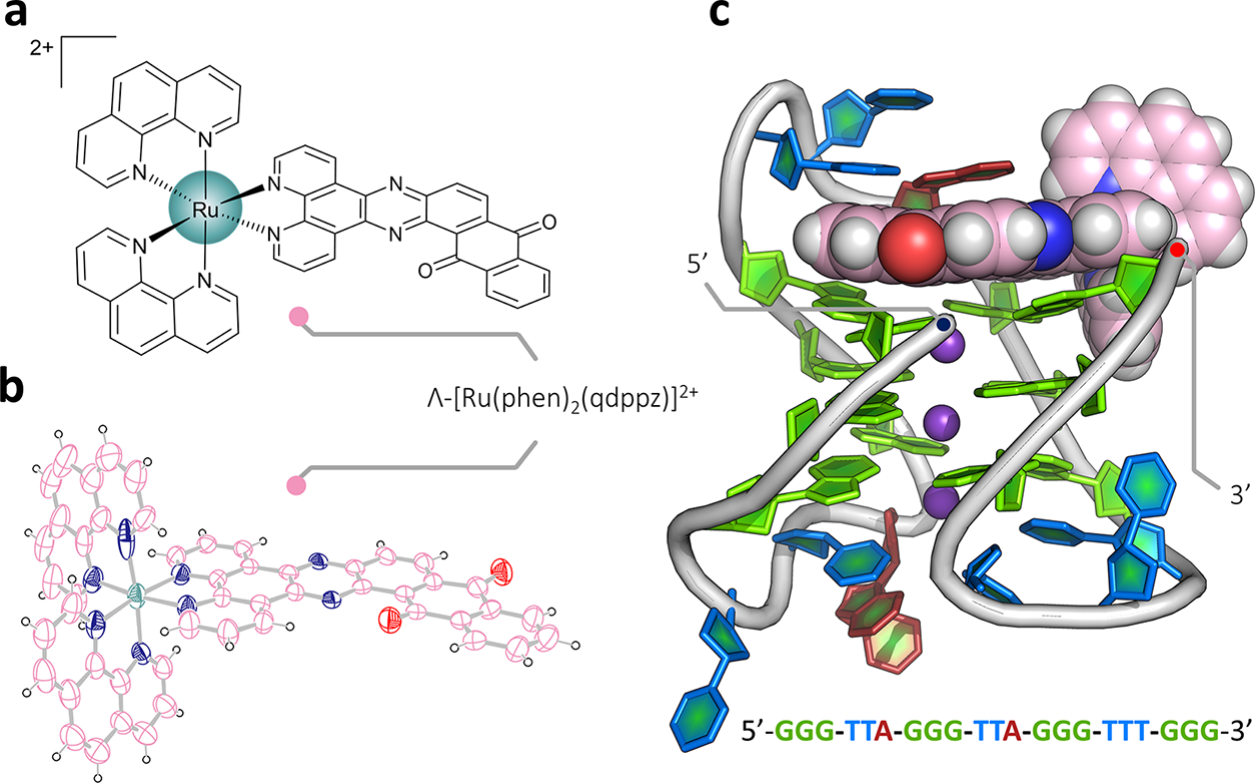
Crystal structures. (a) Line drawing of Λ-(**I**), Λ-[Ru(phen)_2_(qdppz)]^2+^; (b) thermal ellipsoid plot of one cation from the small-molecule structure of rac-(**I**); and (c) overall view of the asymmetric unit of the structure reported here with the DNA sequence shown below. A single strand of the sequence d((G_3_T_2_A)_2_G_3_T_3_G_3_) is found assembled with three K^+^ ions and a single molecule of Λ-(**I**). The Protein Data Bank (PDB) accession code is 7OTB. The color code for residues throughout are as follows: guanine—green, thymine—blue, and adenine—red. Ruthenium complexes are colored in the following scheme; teal for ruthenium, pink for carbon, and dark blue and white for nitrogen and hydrogen, respectively. The complete numbering scheme for Λ-(**I**) is shown in Figure S3.

The ruthenium complex (**I**), shown in Figure 1a, was synthesized by a modified version of a literature procedure^31^ and had previously only been used as the racemic complex, in small-scale studies of binding to calf thymus DNA.^32,33^ The bipyridine analogue, *rac*-[Ru(bpy)_2_(qdppz)]^2+^, has more recently been investigated as a comparative species in the design of complexes exhibiting longer-lived lifetimes for prospective use in energy conversion and photochemotherapy.^34^ We determined the crystal structure of the ruthenium complex alone as the dichloride salt (Figure 1b). For studies with oligonucleotides, the complex was first resolved into enantiomers, and crystallized with the modified telomeric sequence *htel21*T_18_, d((G_3_T_2_A)_2_G_3_TTTG_3_). A clear single-wavelength anomalous dispersion (SAD) map was obtained at the Diamond Light Source. For refinement, we used a 1.44 Å data set measured near the ruthenium absorption edge at 0.5603 Å, refining to a final *R*_work_/*R*_free_ of 0.165/0.181. The complete assembly is shown in Figure 1c, and full details of all the experiments are included in the Supporting Information.

### The description of the structure of the assembly formed between Λ-(**I**) and *htel21*T_18_

**Figure 2:**
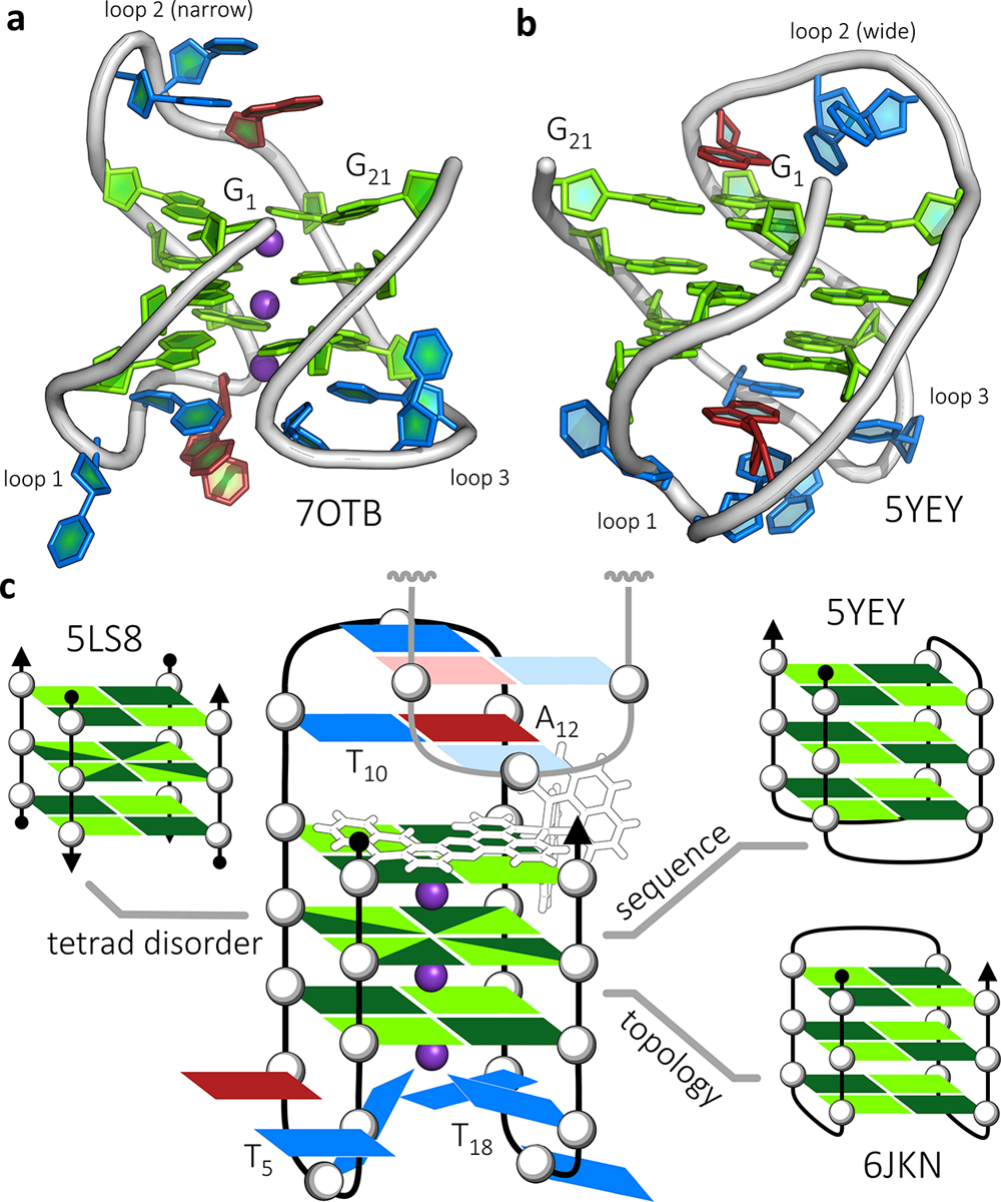
G-quadruplex topologies. (a) Topology of the overall DNA arrangement and three connecting loops of the reported crystal structure (PDB: 7OTB); (b) NMR structure (PDB code: 5YEY) with the same sequence used in this work, which has the opposite strand directionality and loop pattern to that seen here; and (c) schematic of the assembly, with part of the symmetry-related chain that completes it (residues T_10_, T_11_, and T_12_) shown in paler colors. Simplified schematics of antiparallel G-quadruplexes with PDB codes of relevant structural data are shown in the inset, highlighting the structural similarities with related published work. In scheme (c), the *syn*-guanosine conformations are shown in dark green and the *anti* conformations in pale green.

**Figure 3:**
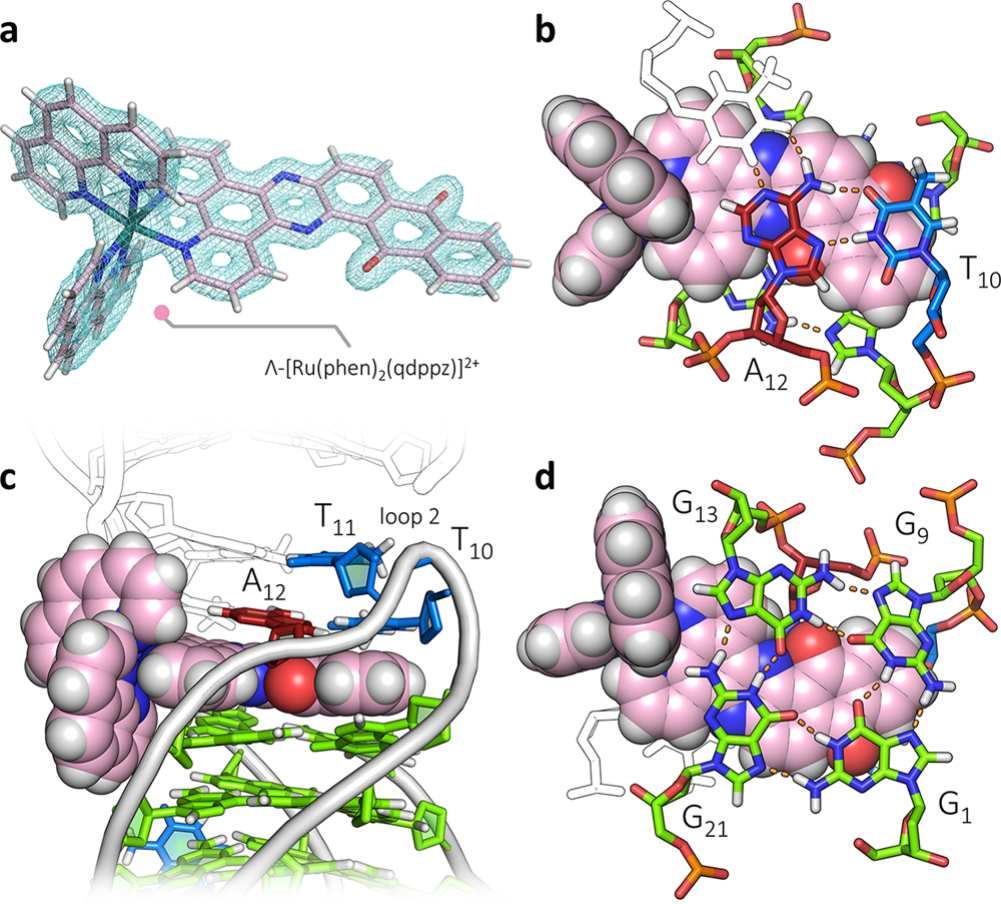
Ligand-binding cavity. (a) Observed electron density around the single copy of Λ-(**I**), contoured at the 1σ (0.29 e Å^–3^) level. (b) Projection showing the triplex formed by T_10_, A_12_ and the symmetry-related T_11_ (in white). The stacking onto the qdppz chromophore is principally with the T_10_–A_12_ Hoogsteen base pair. (c) Side view of the cavity, showing the intercalation of the ligand between the triplex and the quartet, and pointing into the narrow groove. The symmetry-related assembly is in white, with the second ligand omitted for clarity and (d) projection showing the reverse ligand surface and the wide/narrow groove pattern. The qdppz chromophore spans the full width of the G-quartet between the wide grooves.

The overall topology of the DNA chain is shown in Figure 2a. The antiparallel chair has an anticlockwise strand arrangement when placed in the standard reference frame,^35^ to give the opposite overall strand direction from that found in the NMR structure (PDB: 5YEY) shown in Figure 2b.^30^ A schematic of the complete assembly is shown in Figure 2c, including the triplex formation with a symmetry-related strand. This anticlockwise chair has previously been seen only when using 8-bromoguanosine to force a *syn* conformation on G_8_ and G_20_.^36^ Unexpectedly, therefore, this ruthenium complex has stabilized the DNA strand in the anticlockwise strand arrangement, giving the first example of ligand stabilization of the antiparallel chair topology. A key difference between the anticlockwise and clockwise structures is that in the arrangement seen here, the first and third loops bridge the wide grooves of the structure, with the second loop, where the complex is bound, bridging a narrow groove. In the native NMR structure, the first and third loops bridge narrow grooves, and the second loop bridges one of the wide grooves. There are thought to be 14 mechanically possible G4 topologies, of which there are two possible antiparallel chairs. In the standard reference frame, as defined in ref (35), the bound structure has a central narrow loop and is designated as −I_w_ – I_n_ – I_w_. Conversely, the native NMR structure is designated +I_n_ + I_w_ + I_n_, where the positive signs define a clockwise topology (with the subscripts showing the groove widths). The NMR work was performed in 70 mM KCl, whereas our samples were annealed in 10 mM KCl and then transferred to 80 mM NaCl for crystallization. The K^+^-stabilized assembly persisted despite the Na^+^ cation environment. The quality of the final electron density map is shown for the ligand in Figure 3a. The electron density for the whole assembly is shown in Figure S5.

The bound ruthenium complex is notable for its asymmetrically fused anthraquinone-dppz chromophore and its lambda chirality. The extended ligand of the ruthenium complex is bound at one end of the G4 stack in the narrow second lateral loop, and can be thought of as intercalated between a T_10_–A_12_ Hoogsteen base pair and the adjacent G-quartet, as shown in Figure 3b,c. The Ru(phen)_2_ moiety sits in a wide groove, allowing the distal portion of the curved qdppz chromophore to fit the narrow groove created by the G_9_–*syn*T_10_–T_11_–A_12_–*syn*G_13_ loop (loop 2), and stacks onto the G-quartet surface as shown in Figure 3c. There are close contacts between the A_12_ sugar ring and the qdppz ligand, specifically between C2′of A_10_, and C_11_ and C_12_ of the qdppz ligand (Figure S6), and T_10_ adopts an extended *syn* conformation, rare for thymine. The G-quartet adjacent to the extended chromophore is formed by the *syn*G_1_–G_9_–*syn*G_13_–G_21_ base arrangement, with G_13_ and G_21_ being the closest to the Ru atom, forming the wide groove. Inherent in this groove pattern are the alternating *syn* and *anti* guanosine residue conformations.^35^ In this case, the *syn* nucleosides are G_13_ and G_1_, with G_13_ the nucleoside determining the enantiomeric specificity and the base orientation, with a contact between the G_13_ ribose sugar and the adjacent phen ring of the ligand (Figure 3d). The chromophore overlaps with all the four bases of the quartet and shows an unexpected curvature that matches the deviations from planarity in the flanking G-quartet. The footprint of the ancillary phen ligands emphasizes the importance of the lambda chirality, extending over two base pairs in each direction and forming specific contacts in one of the wide grooves, whereas the qdppz ligand spans the wide grooves and is an excellent fit to the G-quartet surface. The qdppz moiety is further integrated into the overall assembly by a network of water molecules centered on the external anthraquinone carbonyl oxygen atom, in addition to the spines of hydration in the narrow grooves. There are limited intermolecular interactions; most notably, the T_10_–A_12_ Hoogsteen base pair formed in loop 2 adds a symmetry-related T_11_, forming a triplex, shown in the schematic of Figure 2c and also in Figure 3b,c.

**Figure 4:**
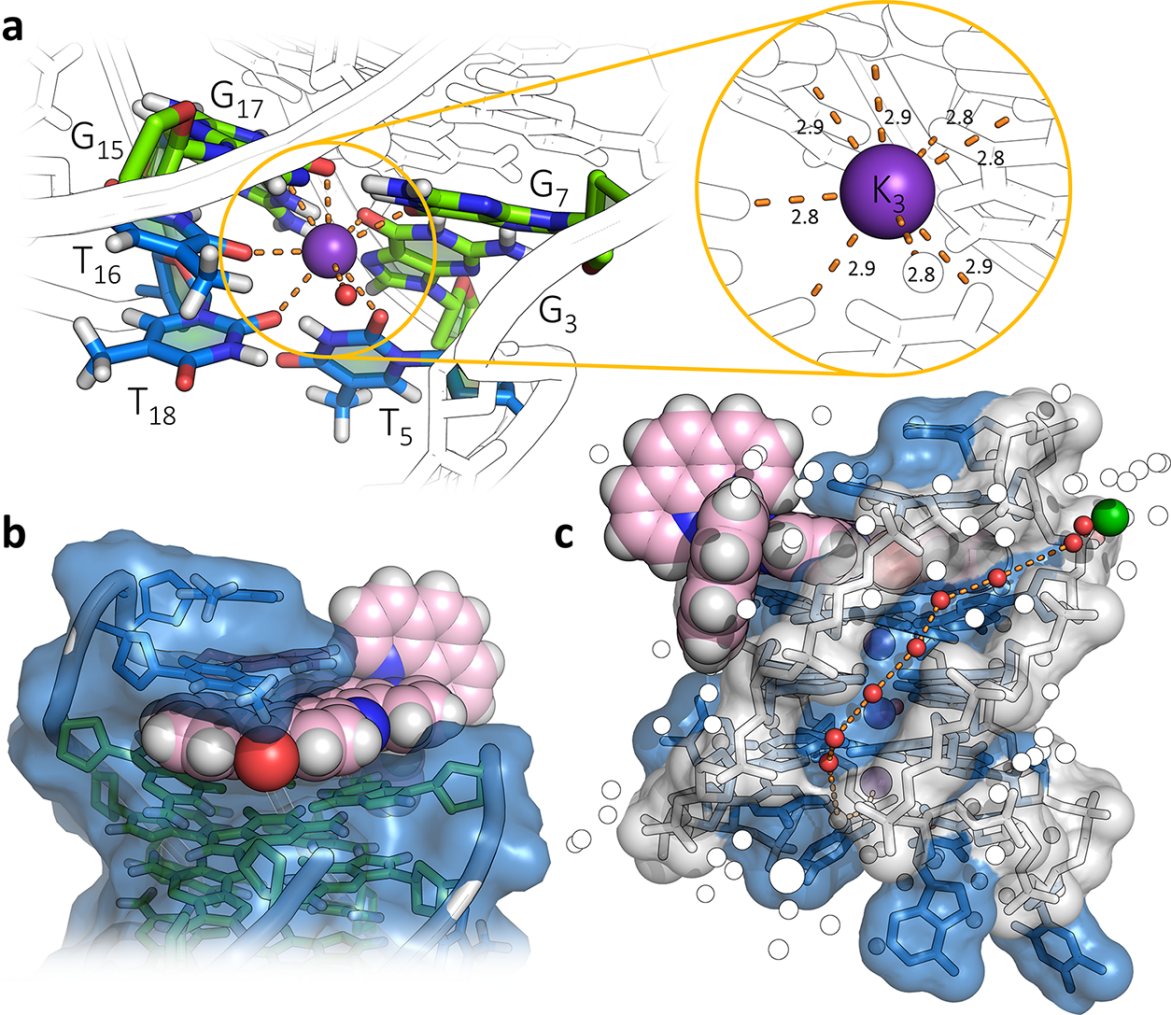
Ligand environments. (a) Environment of K_3_, the K^+^ ion linking the wide loops 1 and 3, coordinating T_5_, T_16_, and T_18_, with the eighth coordination position made up by a water molecule, with the inset showing the consistency of the K–O distances (in Å); (b) binding cavity of Λ-(**I**) highlighting the van der Waals surface of the nucleic acid partially enveloping the complex; and (c) surface of the narrow loop and the narrow groove with the spine of water molecules linking the hydrated Ba^2+^ (green sphere) with the hydrated K^+^ ion K_4_. The environment of the Ba^2+^ ion is shown in Figure S7.

At the other end of the G-quartet stack, anchored by the third K^+^ ion, three thymine residues link the first and third loops (Figure 4a) and are far removed from the ligand cavity (Figure 4b). These interactions, together with extensive structured water, generate the compact globular unit in the crystal (Figure 4c). One advantage of an X-ray structure determination is that the cation assembly holding the G-quartets can be unambiguously determined, with three fully occupied K^+^ ion positions clearly visible in the center of the G-quadruplex assembly, confirming a clear preference for K^+^. There are two typically coordinated K^+^ surrounded by the distorted square antiprism of the guanine carbonyl ligands. The third K^+^ coordinates three thymine carbonyl groups (T_5_, T_16_, and the deliberately introduced variant T_18_) with the eighth coordination position occupied by a water molecule. T_5_ and T_18_ coordinate through the carbonyl oxygen O2, whereas T_16_ coordinates through O4. Notably, the two stabilizing features noted in the NMR structure of the native sequence, a reverse Watson–Crick A_6_–T_18_ base pair and a T_5_–T_16_ hydrogen bond, are both absent. Residue T_17_ is flipped out of the loop and sits in between the phen ligands of an adjacent metal complex. Residues T_4_ and A_6_ are also flipped out, but only A_6_ is disordered and has been fitted into two orientations. Neither makes obvious important intermolecular contacts. Finally, the presence of Ba^2+^ ions was required for successful crystallization, and we see a fully hydrated Ba^2+^ ion in the intermolecular solvent space (Figure 4c and inset to Figure S7). All the eight water molecules of a dodecahedral coordination can be observed. We often find that Ba^2+^, perhaps because it forms [Ba(H_2_O)_8_]^2+^ hydrated ions, is a particularly effective ion for crystallizing ligand–DNA assemblies.^37^

### Geometry of the G-Quartets

**Figure 5:**
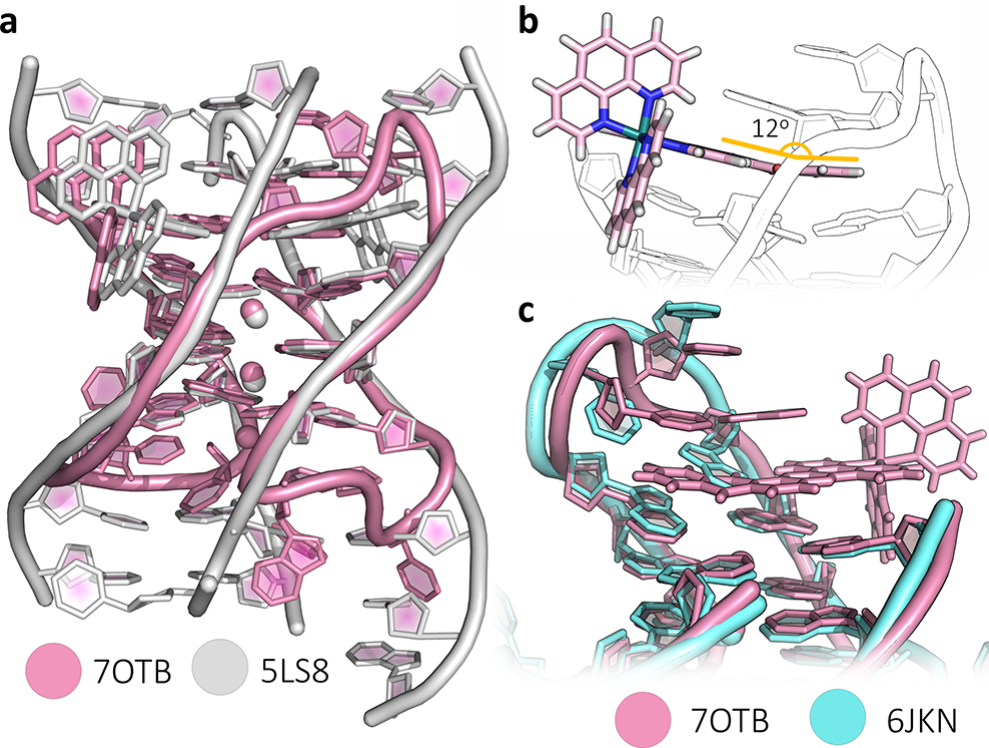
Structural comparisons. (a) Superposition of the structure presented here (pink) with our previously determined structure of [Ru(phen)_2_(11-CN-dppz)]^2+^ bound in 1:1 stoichiometry with the d(TAG_3_T_2_A) octamer (white), with PDB code 5LS8; (b) diagram highlighting the induced fit of the nonplanar qdppz ligand to the similarly nonplanar terminal G-tetrad; and (c) superposition of the structure presented here with the brominated *htel*(8,20-BrG) crystal structure, PDB code 6JKN. The G-quartets show excellent alignment, whereas the second loop shows the realignments to allow intercalation by the qdppz chromophore.

The individual guanosine conformations relate to those of the antiparallel chair topology (Table S4). The electron density map showed clearly that the central quartet should be modeled as a 50:50 mixture of rotations, whereas the flanking quartets clearly have a single rotation (Figure S8). In the NMR structure (PDB code 5YEY), the central quartet was modeled with a single conformation of each residue, as shown in Figure 2d. In that case, to maintain the *syn*-*anti* alternation round the central tetrad, the strand has to have the pattern *syn*G_1_–*anti*G_2_–*anti*G_3_–*syn*G_7_–*syn*G_8_–*anti*G_9_ and so on. The X-ray data require that the pattern becomes *syn*–(*syn*/*anti*)–*anti* for all the four runs of guanines, and in Table S4, we show all possible geometric parameters. In a crystal structure, this is a statistical disorder, with each individual assembly having one of these patterns. If this were an ordered arrangement within the crystal, we would expect there to be two distinct assemblies, with possibly a different crystallographic symmetry. We have previously observed a similar disorder, in the structure of the substituted dppz derivative Λ-[Ru(TAP)_2_(11-CN-dppz)]^2+^ with the octamer d(TAG_3_T_2_A)_4_, which also formed an antiparallel strand arrangement with a similar pattern of alternating *syn* and *anti* conformations and a similar central quartet with a 50:50 mixture of orientations for each residue.^16^ The two structures are shown superimposed in Figure 5a. In that case, the stoichiometry was different, with four Ru complexes per assembly, two stacked at each end of the quartet assembly, and with each lambda enantiomer associated with a *syn*-guanosine residue. We find that these lambda ruthenium complexes stabilize the *syn* conformation, as seen here at *syn*G_13_. The lack of planarity of the G-quartets compared to the curvature of the qdppz suggests some form of induced fit of the ligand, visible in Figure 4b and highlighted in Figure 5b. Here, the curvature follows the base orientations in the adjacent quartet, with a bend of 12°. An inherent property of the antiparallel chair form is the nonplanarity of the bases, so any ligand targeting this topology should have some inherent flexibility. In the case of the qdppz ligand, the principal nonplanar atoms are those of the quinone ring. Remarkably, the antiparallel G4 core geometry also superimposes on the previously crystallized brominated analogue^36^ (Figure 5c).

### Regulation of Replication of the Human Telomeric Sequence

We next examined whether the structure of Λ-(**I**) with this *htel21*T_18_ sequence could be related to the known effects of G4-containing sequences on key biological processes such as replication. In this replication assay, the ability of a G4-binding ligand to impede DNA replication is measured. We therefore compared the inhibitory effects of the enantiomers of complex (**I**) on DNA replication using the Klenow fragment (3′ → 5′ exo−).^38−40^ In this well-developed assay, a time course plotting the rates of the generation of an intermediate stalled replication product followed by a completed full length duplex is compared.^38^ The stabilization of the G-quadruplex by the binding of an appropriate ligand gives a slower rate of the unfolding of the structure, and a detailed interpretation of such data for a range of G4-binding ligands has recently been published.^40^ That work quantified the kinetics and thermodynamics of retardation and made comparisons with several topologies and classes of compounds for which detailed binding models were not available. Because this is a versatile assay and can be run under a wide range of conditions, we were able to carry it out with the modified *htel21*T_18_ sequence since the template strand can be modified to include any G-quadruplex sequence of interest. The normal human telomeric sequence d(T_2_AG_3_)_4_ was therefore compared with the sequence used in this study (see Supporting Information section S1.5 for experimental protocols) with just the single-base modification shown in Figure 1. The enantiomers of the “light-switch” complex [Ru(phen)_2_(dppz)]^2+^ were used as comparative ligands. These complexes bind strongly but relatively nonspecifically to a range of DNA sequences and structures,^41^ but to our knowledge, metal complexes have not previously been studied in this replication assay.

**Figure 6:**
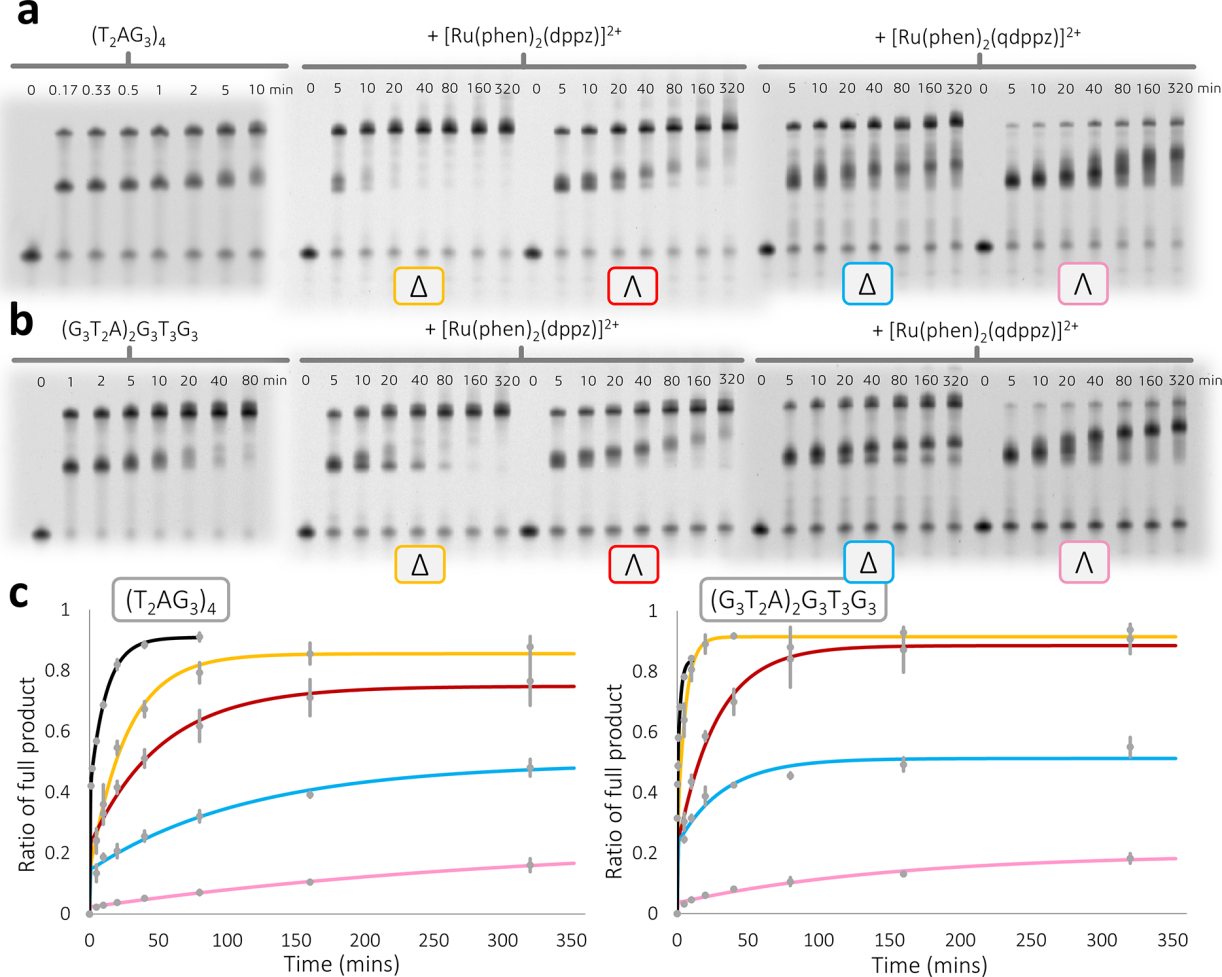
Replication assays. Representative time-dependent denaturing PAGE analyses following the replication of the noncanonical structures formed by (a) (T_2_AG_3_)_4_ or (b) (G_3_T_2_A)_2_G_3_T_3_G_3_ template strands with and without the presence of the enantiomerically pure ligands. Replication experiments were conducted with 10 mM KCl at pH 6.5 (see Supporting Information section S1.5 for complete experimental details). (c) Kinetic analysis of the time-course of each experiment showing a comparison of each ligand by its ability to stall the replication of the given template sequence. The error bars shown are calculated as the s.d. of triplicate experiments (see Figures S10 and S11 for triplicates and SYBR-stained gels). Calculated rate constants for all the systems investigated can be found in Table S5.

All the assays were carried out in triplicate (Figures S10–S13 show full details). Figure 6 shows the results of the experiment when the G4-forming sequence in the template was separated from the remaining randomized sequence by 15 thymine residues to guard against unexpected hairpin formation and other artifacts. In the absence of any ligand in this assay, the stalled product formed as the enzyme encounters the G-quadruplex disappears within a few minutes, as shown by the gel assays. The rate differences between the standard and T_18_-modified human telomeric sequences were small, so the single A–T substitution has only a limited effect on the kinetics of polymerization in this system. The results showed that Δ-[Ru(phen)_2_(dppz)]^2+^ was clearly the least effective compound, and Λ-(**I**) was the most effective. Even after 320 min of reaction in the presence of Λ-(**I**), only ∼15% of the product was observed in the polyacrylamide gel electrophoresis (PAGE) analysis. Furthermore, in the presence of LiCl, a known destabilizer of secondary structure, Λ-(**I**) was still able to retard the polymerase action (Figures S12 and S13), suggesting that the complex could stabilize the G4 structure without K^+^. We have recently investigated a library of known G-quadruplex-binding compounds for their ability to stall the replication of d((T_2_AG_3_)_4_) in an analogous fashion to the experiments conducted here. It was established that the naphthalene diimide derivative, cNDI1-py, generated by far the strongest stalling effect on the replication of the quadruplex, where after 320 min of reaction, approximately 20% of the full-length product was observed. Considering that this inhibition was also observed at a 10-fold higher potassium concentration (100 mM K^+^ in that work), it is fair to say that Λ-(**I**) has exhibited the highest potency we have seen to date with this assay.^40^ This observation, seen with both sequences, strongly suggests a highly specific binding mode, as implied by the crystal structure.

We then checked, using circular dichroism, whether there was any evidence for a clear relationship between the kinetics of replication and the thermodynamic stability as shown by melting temperatures. Figures S14 and S15 show the thermal denaturation profiles of each studied system, and Table S5 summarizes the observed melting temperatures alongside the kinetic data. We found no direct relationship to the kinetics of replication stalling. The result is consistent with our recent findings on specific G4 topology and ligand interactions.^40^

## Discussion

Studies of ligand binding to the human telomeric sequence by X-ray crystallography have all given a parallel topology, as shown by the most recent examples.^42−46^ In most cases, crystallization required the addition of extra bases to the minimal *htel21* sequence, but not always.^42^ In our hands, none of these strategies was successful, either with Λ-(**I**) or with other ruthenium compounds. We conclude that ligands that bind to the parallel topology can also facilitate crystallization. Such ligands have large flat central surfaces and flexible sidechains, such as the naphthalene diimide family.^42^ A ligand such as Λ-(**I**), which has a rigid three-dimensionality and cannot be stacked in a crystal, has finally been cocrystallized with *htel21*T_18_ using the approach described in this paper.

**Figure 7:**
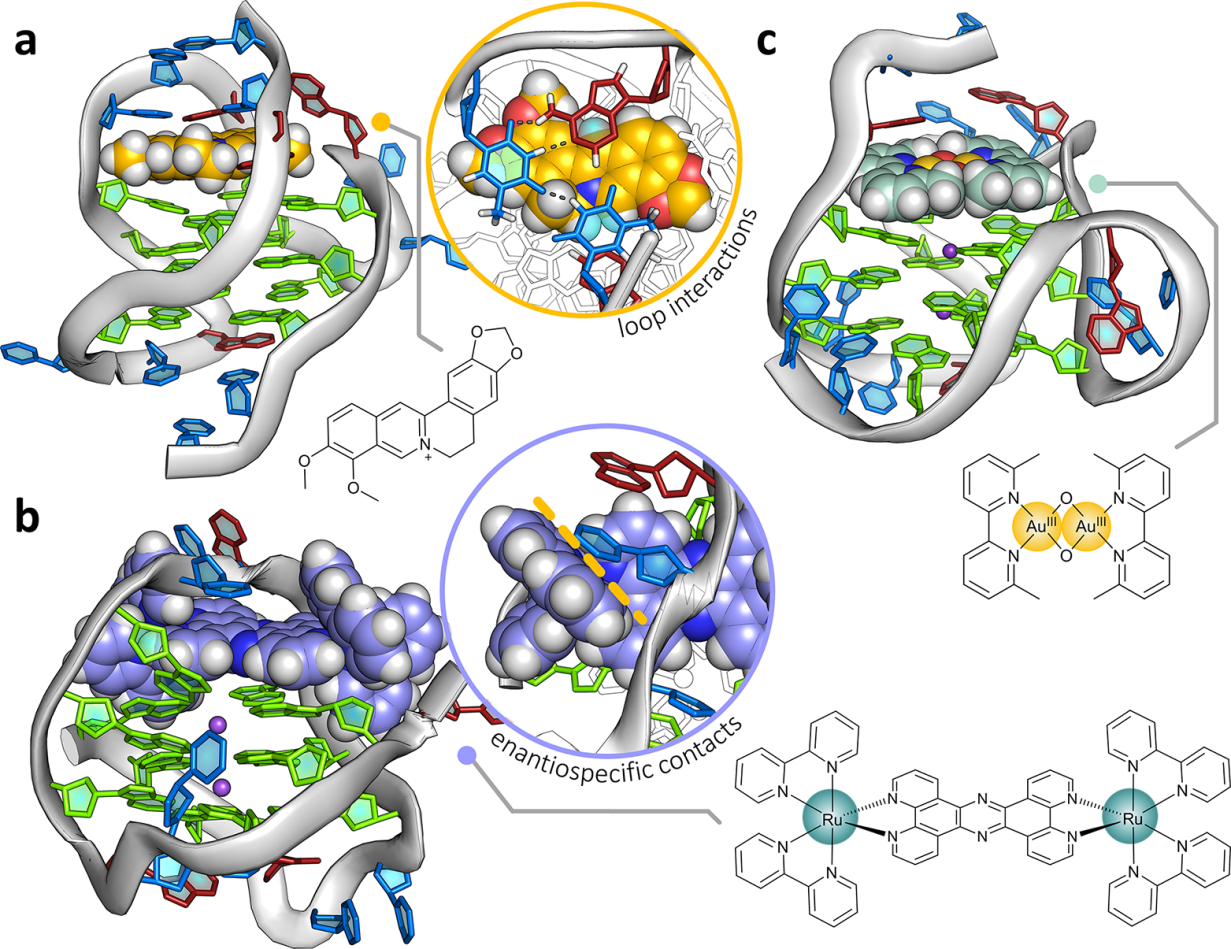
NMR structural comparisons. Structures obtained via NMR studies of ligand binding to G-quadruplexes that exhibit comparative structural features or ligand–DNA interactions. (a) Epiberberine bound to the hybrid-2 form of *htelo26*, highlighting the pseudo-intercalation between a terminal G-tetrad and the nucleobases in the second loop. (b) ΛΛ-[{Ru(bpy)_2_}_2_(tpphz)]^2+^ bound via a loop-threading mode to the antiparallel form of *htelo*22. The complex binds with diastereoselectivity and exhibits complex–nucleobase interactions that are enantiospecific to the lambda isomer (inset), similar to what is observed in the reported structure. (c) Au-oxo6 bound to the hybrid-2 form of *htelo*26, (TTAGGG)_4_TT. This structure shows the gold complex neatly end-capping a terminal G-tetrad, but also interacting via π-stacking with an adenine in the flanking region.

The use of Λ-(**I**) has given the first demonstration that an antiparallel G4 topology can be crystallized with a G4-binding ligand. Of the 29 reported X-ray structures of G4–ligand binding, all the unimolecular complexes have parallel topology, including those derived from the human telomeric sequence. The only antiparallel or hybrid topology studies of ligand binding to the human telomeric sequence have come from NMR. There are nine examples (Table S6), with three illustrated in Figure 7. The two antiparallel basket structures derive from the closest ligands to those reported here, two bis-ruthenium complexes. The antiparallel basket topology forms in Na^+^ solution, showing enantiospecific threading through the central diagonal loop.^23^ Octahedral coordination drives binding specificity, as here. The closest comparison with the present work is the stabilization of the hybrid-2 topology by the ligand epiberberine.^12^ In that example, we see the stacking of T_13_ and A_15_ from the second loop onto the chromophore, without base-pairing. The ligand is coplanar with A_3_, creating a multilayer binding pocket not previously observed. The hybrid-2 topology is also stabilized by the binuclear gold(III) complex shown, a very neat fit to the G-quartet surface and stacking onto the flanking adenine, but without any additional loop interactions.^47^

The replication assay used showed a strong inhibition of replication by both the native *htel21* sequence and the *htel21*T_18_-modified sequence. Previous studies from the Sugimoto laboratory have correlated the unfolding rate in this assay with the presumed G4 topology.^38^ In this study, although it seems plausible to assume the antiparallel topology is stabilized by Λ-(**I**), there is also strong inhibition by the Δ-enantiomer, and we can draw no definite conclusion about the topology with this enantiomer. Similarly, our previous crystallographic studies with Λ-[Ru(TAP)_2_(dppz)]^2+^ and the short G4-forming sequence d(TAGGGTT) did not show any binding to the G4 surface.^26^ Rather, this study showed several metal complexes enmeshed among the terminal adenine and thymine bases. For both *htel21* and *htel21*T_18_, the pattern of inhibition by the four complexes chosen is very similar. We conclude from this that the effect of the single-base modification has little influence on the stability of the metal complex-G4 interaction. The replacement of the adenine base by thymine at T_18_ does not change the way that Λ-(**I**) interacts with the T_10_–T_11_–A_12_ loop, as judged by the replication assay, and our tentative assumption is that this compound drives both sequences to the antiparallel chair form seen in the crystal structure.

## Summary

For this study, we chose *htel21*T_18_ specifically because it formed the antiparallel chair structure under solution NMR conditions and with little evidence for any solution heterogeneity. What is striking and unexpected is the reversal of chirality of the nucleic acid strand while retaining the antiparallel chair topology. The specific intercalation of Λ-(**I**) into a narrow loop formed by TTA has no precedent in the examples mentioned above. Furthermore, the powerful inhibition by Λ-(**I**) in the replication assay reported here relates the crystal structure to solution behavior. The replication assay has allowed us to make a comparison between the enantiomers of (**I**) and the two telomeric sequences. Λ-(**I**) gave the strongest inhibition of the four ruthenium complexes, but also when compared to the disparate group of G4-binding ligands previously studied.^40^ This study allows us to correlate a very specific topology of binding with a very powerful kinetic effect on replication in the presence of a G4-forming DNA sequence. It points the way to a new class of topologically selective G-quadruplex-binding ligands.

## Supporting Information Available

Synthetic procedures, NMR spectra and other characterization, crystal structure data, and replication kinetics (PDF)

## Accession Codes

CCDC 2090875 contains the supplementary crystallographic data for this paper. These data can be obtained free of charge via www.ccdc.cam.ac.uk/data_request/cif, or by emailing data_request@ccdc.cam.ac.uk, or by contacting The Cambridge Crystallographic Data Centre, 12 Union Road, Cambridge CB2 1EZ, UK; fax: +44 1223 336033.
