# Supplementary material for: Ruthenium Polypyridyl Complex Bound to a Unimolecular Chair-Form G-Quadruplex
Source: J Am Chem Soc. 2022 Mar 24;144(13):5956–64. doi: 10.1021/jacs.2c00178 (PMC8991003; doi:10.1021/jacs.2c00178)
Supplement: Supplementary file 1 — ja2c00178_si_001.pdf [file ja2c00178_si_001.pdf]

Supporting Information

Ruthenium Polypyridyl Complex Bound To A Unimolecular Chair-Form G-quadruplex

Kane T. McQuaid,*[a] Shuntaro Takahashi,[b] Lena Baumgaertner,[a] David J. Cardin,[a] Neil G. Paterson,[c] James P. Hall,[d] Naoki Sugimoto,[b,e] and Christine J. Cardin[a]

[a] Department of Chemistry, University of Reading, Whiteknights, Reading, Berkshire, RG6 6AD, UK
[b] Frontier Institute for Biomolecular Engineering Research, Konan University, Kobe, Japan, 650-0047
[c] Diamond Light Source Ltd., Harwell Science and Innovation Campus, Didcot, Oxfordshire, OX11 0DE, UK
[d] Department of Pharmacy, University of Reading, Whiteknights, Reading, Berkshire, RG6 6AP, UK
[e] FIRST (Graduate School of Frontiers of Innovative Research in Science and Technology), Konan University, 7-1-20 Minatojima-Minamimachi, Chuo-ku, Kobe 650-0047, Japan

Email: k.mcquaid@reading.ac.uk, or c.j.cardin@reading.ac.uk

Contents

1. Experimental 1

1.1. Materials 1

1.2. Instrumentation 1

1.3. Synthesis 1

1.3.1. 12,17-dihydronaphtho[2,3-h]dipyrido[3,2-a:2′,3′-c]phenazine-12,17-dione (qdppz) 1

1.3.2. Ruthenium bis-(phenanthroline) 12,17-dihydro-naphthodipyridophenazine-12,17-dione dichloride (*rac*-[Ru(phen)$_2$(qdppz)]Cl$_2$) 2

1.4 Enantiomeric Resolution 3

1.5 X-ray Crystallography 3

1.5.1 Λ-[Ru(phen)$_2$(qdppz)]·Cl$_2$ 3

1.5.2 Λ-[Ru(phen)$_2$(qdppz)]$^{2+}$ with d(GGGTTAGGGTTAGGGTTTGGG) 4

1.6 Klenow Fragment Replication 4

1.6.1 Klenow fragment (exo$^-$) preparation 4

1.6.2 Klenow fragment replication assay 5

1.7 Circular Dichroism 5

2. Supplementary Figures and Tables 6

2.1. Figure S1 – ^{1}H NMR Spectra 6

2.2 Figure S2 – HPLC chromatograms (a and b) and CD spectra (c and d) of Λ/Δ-[Ru(phen)$_2$(dppz)]$^{2+}$ and Λ/Δ-[Ru(phen)$_2$(qdppz)]$^{2+}$, respectively 7

2.3 Table S1 - Data collection and processing parameters/refinement results for the small molecule dataset 8

2.4 Figure S3 – ORTEP model of the crystal structure of Λ-[Ru(phen)$_2$(qdppz)]$^{2+}$ 9

2.5 Table S2 – Selected bond, angles, and torsions from the small molecule dataset 9

2.6 Figure S4 – Perspective views of the unit cells of the small molecule dataset 10

2.7 Table S3 – Collection and processing statistics of 7OTB 11

2.8 Table S4 – Conformational analysis of 7OTB 12

2.9 Figure S5 – Asymmetric unit of 7OTB 14

2.10 Figure S6 – Ligand-adenine close contact 14

2.11 Figure S7 – Water/H-bonding network of 7OTB 15

2.12 Figure S8 – Central G-tetrad syn/anti order 16

2.13 Figure S9 – Superimposition of 7OTB and 6JKN 17

2.14 Figure S10 – Replication Analysis of (T$_2$AG$_3$)$_4$ 18

2.15 Figure S11 – Replication Analysis of $(G_3T_2A)_2G_3T_3G_3$ 19

2.16 Figure S12 – Replication Analysis of $(T_2AG_3)_4$ in LiCl 20

2.17 Figure S13 – Replication Analysis of $(G_3T_2A)_2G_3T_3G_3$ in LiCl 20

2.18 Table S5 – Kinetic analysis of the replication assays 21

2.19 Figure S14 – CD Thermal Melts of $(T_2AG_3)_4$ in the presence of ligands 22

2.20 Figure S15 – CD Thermal Melts of $(G_3T_2A)_2G_3T_3G_3$ in the presence of ligands 23

2.21 Figure S16 - CD melting profiles of $(T_2AG_3)_4$ and $(G_3T_2A)_2G_3T_3G_3$ in the presence of ruthenium complexes 24

2.22 Table S6 Published antiparallel G-quadruplex structures 25

3. References 25

1. Experimental

1.1. Materials

Oligonucleotides were purchased from Eurogentec Ltd. as double HPLC-purified solids and were used without further purification. Unless otherwise stated, all other materials and chemicals were sourced from Sigma Aldrich or Honeywell research chemicals. Sephadex C-25 anion exchange stationary phase and Dowex 1X2 Chloride form anion exchange resin were purchased from GE Healthcare. All solvents, unless stated in the experimental, were obtained at HPLC grade and used without further purification. Where further purification was needed, protocol from "Purification of Laboratory Chemicals, 4th edition, Armarego *et. al.*" was followed. Deuterated solvents for NMR analysis were purchased either through Sigma-Aldrich or Cambridge Isotope Laboratories.

1.2. Instrumentation

Unless otherwise stated, all ^{1}H NMR spectra were collected on a Bruker Nanobay 400 MHz instrument, with the majority of ^{13}C NMR spectra collected on a Bruker DPX 400.1 MHz machine operating at 100.1 MHz. Both machines were calibrated against a tetramethylsilane (TMS) internal standard and have two channels running TOPSPIN 2.4 and ICON NMR 4.2. All *J*-coupling constants were reported following normalisation against the used Larmor frequency, where a few couplings were omitted subject to spectral resolution.

High resolution ESI mass spectra were recorded on a Thermo Scientific LTQ Orbitrap XL running in positive ion mode. Fragmented Ions were detected on an Orbitrap Ion trap photodiode array detector and were determined via peak matching against the internally calibrated lock mass for Diisooctyl phthalate (*m/z* = 413.26623 Da). Data analysis was performed on the Xcalibur Qual Browser software package and all accurate masses are reported within 3 ppm.

1.3. Synthesis

1.3.1. 12,17-dihydronaphtho[2,3-h]dipyrido[3,2-a:2',3'-c]phenazine-12,17-dione (qdppz)

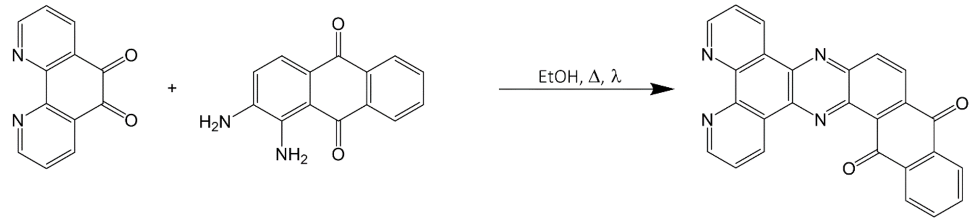

Phendione (50 mg, 0.238 mmol) and 1,2-diaminoanthraquinone (56.7 mg, 0.238 mmol) were both suspended together in an ethanolic solution (7 mL) containing a trace amount of *p*-toluenesulfonic acid within a CEM microwave tube (10 mL). The violet coloured solution was degassed/evacuated with Ar for 15 minutes before being fully sealed and installed into the synthetic microwave. The sample was irradiated with 150 W at 140 °C for 20 minutes, yielding a deep red/violet solution which was ensured to be cool and then filtered *in vacuo* to collect the black precipitate. The powder was suspended in hot chloroform (100 mL) in the presence of powdered charcoal and filtered through a glass frit, yielding a brown solution. The filtrate was reduced to approximately 5 mL in volume (mixture of purple and yellow

coloured precipitation is noted) and diethyl ether (50 mL) was added to complete the precipitation. The powder was collected via suction filtration and washed with diethyl ether (3 x 10 mL), and the target product as yellow-ochre powder (52 mg, 0.129 mmol, 54 %).

δ_H (400 MHz, TMS, $CDCl_3$-d) – 9.90 (dd, *J* = 8.1, 1.6 Hz, 1H), 9.66 (dd, *J* = 7.6, 1.8 Hz, 1H), 9.34 (d, *J* = 4.7 Hz, 2H), 8.84 (d, *J* = 8.8 Hz, 1H), 8.72 (d, *J* = 8.8 Hz, 1H), 8.44 (d, *J* = 7.6 Hz, 1H), 8.35 (d, *J* = 7.6 Hz, 1H), 7.96-7.88 (m, 2H), and 7.90-7.82 ppm (m, 2H).

HRMS-ES (m/z) – Found ($[M+H]^+$, 413.1032); calc. 413.1033 ($C_{26}N_4O_2H_{13}^+$).

1.3.2. Ruthenium bis-(phenanthroline) 12,17-dihydro-naphtho-dipyridophenazine-12,17-dione dichloride (*rac*-$[Ru(phen)_2(qdppz)]Cl_2$)

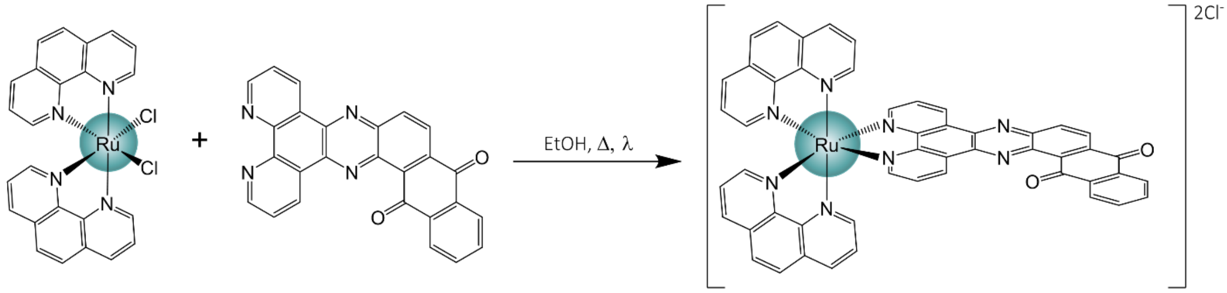

Ru(phen)$_2$Cl$_2$ (81 mg, 0.15 mmol) and qdppz (62 mg, 0.15 mmol) were both suspended together in an aqueous ethanol solution (7 mL, 1:1) within a CEM microwave tube (10 mL). The violet coloured solution was degassed/evacuated with Ar for 15 minutes before being fully sealed and installed into the microwave. The sample was irradiated at 140W at 60°C for 40 minutes, yielding a deep red/brown solution which was cooled and then filtered *via* suction filtration. Subsequent precipitation of the target compound from the filtrate was achieved by metathesis via dropwise addition of a saturated solution of aqueous potassium hexafluorophosphate (KPF_6). Isolation of the PF_6^- salt by *in vacuo* filtration yielded a dark orange/brown solid, which, after washing with cold water (2 x 2 mL) was allowed to dry in air. The complex was then further purified via flash chromatography on silica using 80:15:5 $CH_3CN/H_20/KNO_3$ as the mobile phase (eluting as a deep orange/red band). The collected aliquots were combined and desalted via metathesis to the hexafluorophosphate salt and finally treated with Amberlite resin (IRA-400, Cl^- form, 2.4 g) after being dissolved in a minimum amount of 2:1 H_2O/ACN, to yield the complex as a dark red microcrystalline solid (68. 2 mg, 0.072 mmol, 62 %).

δ_H (400 MHz, CD_3CN-*d_3*) – 9.76 (dd, *J* = 8.2, 1.3 Hz, 1H), 9.64 (dd, *J* = 8.2, 1.3 Hz, 1H), 8.88 (d, *J* = 8.9 Hz, 1H), 8.81 (d, *J* = 8.9 Hz, 1H), 8.66 (ddt, *J* = 8.1, 4.5, 1.3 Hz, 4H), 8.64 (dd, *J* = 5.4, 1.3 Hz, 1H), 8.33 (dd, *J* = 7.7, 1.3 Hz, 1H), 8.31 (d, *J* = 2.7 Hz, 4H), 8.29 (m, 3H), 8.19 (td, *J* = 5.1, 1.3 Hz, 2H), 8.06 (dd, *J* = 5.2, 1.3, 2H), 7.99 (td, *J* = 7.5, 1.5 Hz, 1H), 7.93 (td, *J* = 7.5, 1.5 Hz, 1H), 7.87 (dd, *J* = 8.2, 5.4 Hz, 1H), 7.81 (dd, *J* = 8.2, 5.4 Hz, 1H), 7.72 (ddd, *J* = 8.3, 5.2, 4.4 Hz, 2H), and 7.68 ppm (dd, *J* = 8.3, 5.2 Hz, 2H). *See Figure S1*.

δ_C (401 MHz, CD_3CN-*d_3*) – 183.75, 183.16, 155.59, 155.44, 153.91, 153.89, 153.60, 153.58, 152.14, 151.93, 148.46, 148.37, 144.78, 142.83, 141.28, 140.87, 137.64, 137.61, 137.57, 136.32, 135.65, 135.41, 134.62, 134.48, 134.23, 132.46, 131.71, 131.68, 131.66, 128.78, 128.72, 128.70, 128.67, 128.19, 127.97, 127.36, 126.98, 126.56, and 126.51 ppm.

HRMS-ES (m/z) – Found (M^+, 437.0691); calc. 437.0684 ($RuC_{50}N_8O_2H_{28}^{2+}$)

1.4 Enantiomeric Resolution

Semi-preparative chiral resolution of the racemic complexes, $[Ru(phen)_2(dppz)]^{2+}$ and $[Ru(phen)_2(qdppz)]^{2+}$ was achieved using a Hitachi Primeaide HPLC arrangement equipped with a CF6 LARIHC cyclofructan based chiral column (internal dimensions; 10 x 250 mm) supplied by AZYP separations LLC (Arlington, Texas, US). Successful baseline separations were achieved using a MeOH:ACN:TEA:AA mobile phase; with the anthraquinone enantiomers separated using a ratio of 60:40:2:0.8, whereas the phenanthroline parent complex separated at a ratio of 60:40:4:1.6. Analysis was performed at a flow rate of 5 mL min^{-1} where each preparative injection was 200 µL in volume with an analyte concentration of 3 mg mL^{-1}. Eluent fractions were combined in centrifuge tubes and to each, 2 mL of a 200 mM aqueous solution of potassium hexafluorophosphate was added. The solutions were then reduced under pressure using a DNA concentrator at 40 ^{o}C for 24 hrs to remove the organic eluent. Following metathesis, the precipitate was collected via suction filtration and washed with fractions of HPLC water (5 x 5 mL). The water-soluble chloride salts were then prepared by dissolving the enantiomers in 60:40 H_2O:ACN and stirring the solutions overnight in the presence of excess Amberlite IRA-400 anion exchange resin (Cl^- form). Complex purity was verified by electrospray mass spectrometry (ES-MS); optical purity was first confirmed by analytical chiral HPLC to quantify the quality of the enantiomeric excess of each work up (in each case EE was >99 % by peak area). Circular dichroism of the aqueous solutions (Figure S2) confirmed the success of the separations.

1.5 X-ray Crystallography

1.5.1 Λ-$[Ru(phen)_2(qdppz)]\cdot Cl_2$

1.5.1.1 Crystallisation Parameters

Crystals suitable for X-ray diffraction experiments were obtained by the vapour diffusion of diethyl ether into a saturated solution of Λ-$[Ru(phen)_2(qdppz)]\cdot Cl_2$ in acetonitrile. Dark Red/Brown Rods grew after approximately 2 weeks at 277 K.

1.5.1.2 Data Collection and Structure Solution

Data collection was performed on a Rigaku Synergy-S diffractometer with images collected on a HyPix-6000 pixel detector. Cu Kα radiation (λ = 1.5418 Å) was used on a crystal fragment with approximate dimensions 50x50x200 µm that was cooled to 100 K using a stream of nitrogen gas. Data collection, reduction, and processing was performed using the CrysAlisPro program package. SHELXT was utilised in the Olex2 software suite to solve the structures and they were refined against F2 using a full-matrix least-squares minimisation in SHELXL, All non-hydrogen atoms were refined anisotropically, and hydrogen atoms were first placed in calculated positions and refined using a riding model. Data collection and final refinement statistics are shown in Table S1. An Ortep model is shown in Figure S3, with selected bond lengths and angles given in Table S2, and unit cell contents are shown in Figure S4. Experimental data and refined structures were uploaded to the IUCr checkCIF server and no warnings above level C were reported. The structure is deposited in the CCDC CSD under the refcode 2090875.

1.5.2 Λ-[Ru(phen)$_2$(qdppz)]$^{2+}$ with d(GGGTTAGGGTTAGGGTTTGGG)

1.5.2.1 Crystallisation Parameters

Crystals containing the oligonucleotide d(GGGTTAGGGTTAGGGTTTGGG) and the ruthenium complex [Ru(phen)$_2$(qdppz)]$^{2+}$ were grown from sitting drops via vapour diffusion of water at 277 K. Crystals suitable for X-ray diffraction experiments were obtained from a solution formed by the addition of following two constitutions; 1μL of a pre-annealed mixture of the single stranded oligonucleotide at 200 μM with the complex Λ-[Ru(phen)$_2$(qdppz)]·Cl_2 at 200 μM in a 10 mM KCl buffer; and 1 μL of a solution containing 80 mM sodium chloride, 20 mM barium chloride dihydrate, 12 mM spermine tetrahydrochloride, and 40 % v/v 2-methyl-2, 4-pentanediol (MPD); all buffered to pH 7.0 using 40 mM sodium cacodylate trihydrate. The sitting drop was equilibrated against 100 μL of 40 % v/v MPD in H_2O forming dark orange/red hexagonal rods within 4 days of preparation.

1.5.2.2 Data Collection and Structure Solution

The data were collected at Diamond Light Source Ltd., on beamline I03 using radiation with a wavelength of 0.5604 Å from a flash cooled crystal at 100 K. 360° of data were collected with an oscillation of 0.1^{o} per frame, generating 3600 images. The resulting data were processed using XDS[1] and XSCALE[2] through the xia2[3] pipeline, finding 11038 unique reflections to a resolution of 1.44 Å and an overall I/σ of 10.9. A separate dataset collected on the same crystal using radiation with a wavelength of 1.771 Å was used to build an initial substructure using the anomalous scattering of ruthenium/barium by single wavelength anomalous dispersion (SAD) methodology using the Phaser-EP pipeline in the PHENIX software package.[4,5] This substructure was used to calculate an initial phasing solution for the higher resolution dataset. The crystallographic model was built using WinCoot[6] and refined using Phenix.refine[7] to give a final R_{work} of 0.1648 and an R_{free} of 0.1810 reserving 10 % of the total reflections for the R_{free} set. Table S3 highlights the data collection(s) and refinement statistics; the structure is deposited in the Protein Data Bank with PDB accession ID: **7OTB**. Full DNA conformational analysis was conducted using a combination of programs, namely the DNATCO v3.2 and w3DNA servers.[8,9] Figures were produced using the PyMOL software suite.

1.6 Klenow Fragment Replication

1.6.1 Klenow fragment (exo$^-$) preparation

The Klenow Fragment (KF) encoding gene was amplified from *E. coli* genomic DNA (JM109) *via* polymerase chain reaction (PCR). PCR was proceeded using PrimeSTAR DNA polymerase (Takara Bio) and primers (5’-GGGACCATATGGTGATTTCTTATGACAACTACG-3’ and 5’-GGGAGAATTCTTAGT-GCGCCTGATCCCAG-3’) sourced from Eurofin Genomics (Japan). Following digestion using NdeI and EcoRI, the cloned DNA fragments were cloned into pMal-p5x vector (New England Bio Labs). KF exo$^-$ (D355A, E357A) was prepared by mutating the constructed plasmid using a QuikChange mutagenesis kit (Stratagene) followed by using the mutated vector to transform *E. coli* EG2523 (New England Bio Labs). The cells were then cultured in Luria-Bertani medium containing ampicillin and worked up to an A_{600} of roughly 0.5 followed by addition of isopropyl β-*D*-1-thiogalactopyranoside (IPTG). The cultured cells were harvested and lysed via sonication and the soluble fraction was loaded onto an amylose resin packed column (New England Bio Labs). Following treatment with Factor Xa protease to eliminate the MBP-tag, the KF exo- was purified over a Hitrap Heparin column followed by purification through a Hiload Superdex 200 (GE Healthcare). The purified KF exo- was dialysed against a dialysis buffer containing 50 mM Tris-HCl (pH 7.2), 1 mM EDTA, 1 mM DTT, 100 mM NaCl and 50 % glycerol.

Concentration was determined spectrophotometrically using an extinction coefficient of 58,790 $M^{-1}cm^{-1}$ at 280 nm. The mutant enzyme was stored at -30 °C until use.

1.6.2 Klenow fragment replication assay

Template strand (5'-TTAGGGTTAGGGTTAGGGTTAGGGTTTTTTTTTTTTTTTCCTATAGTGAGTCGT-ATTACCC-3' or 5'-GGGTTAGGGTTAGGGTTTGGGTTTTTTTTTTTTTTTTCCTATAGTGAGTCGTATTACCC-3') and primer strand (5'-FAM-GGGTAATACGACTCACTATAGG-3') oligonucleotides were annealed in the following buffer and in the presence, if quoted, of the necessary ligand: 10 mM Tris-HCl (pH 7.5), 8 mM $MgCl_2$, 1 µM FAM-labelled primer DNA, 1 µM template DNA, 10 µM ligand, 250 µM dNTPs and 10 mM KCl (or LiCl where indicated). After annealing (95 °C to 4 °C at -1 °C min^{-1}), the mixture was incubated at 37 °C and a 100 µM solution of KF exo- was added to the reaction mixture (final concentration of 1 µM) to initiate the enzymatic reaction. At given time intervals during the reaction, aliquots of the mixture were quenched using a stopping solution containing 10 mM EDTA and 80 wt% formamide. Reaction products were separated by denaturing polyacrylamide gel electrophoresis (PAGE) using gel containing 8 M urea in a TBE buffer at 70 °C for 1 hour at 200 V. Alongside product lanes, a molecular weight marker DNA ladder (10-bp) and a bromophenol blue running aid were run in adjacent and terminal lanes. Gel images were captured using a Fujifilm Fluoreimager FLA-5100 utilising a laser excitation wavelength of 473 nm. Images were collected before and after staining with SYBR Gold (ThermoFisher Scientific) to highlight unlabelled products. Band intensities were analysed using ImageJ2 software package (NIH) by quantifying peak areas after baselining the necessary lanes. The reaction yield of full-length product (*P*) was quantified by calculating the ratio of intensity of the full-length product bands to the aggregate intensity of all bands. Reaction rate analysis was performed using Dynafit software package (Biokin) after evaluating a global fit of the reaction. This was achieved by applying a kinetic model to the following two-step sequential model;

$$P_0 \overset{k_s}{\rightarrow} P_s \overset{k_f}{\rightarrow} P_f$$

Where P_0 is the starting state of the reaction, P_s represents the state immediately after unwinding of the reaction stall (motif), P_f represents the state after the replication of the full-length product is completed; and k_s and k_f are the rate constants that define the rate of reaction between states.

1.7 Circular Dichroism

Circular dichroism spectra were collected at 37 °C on a JASCO J-1500 CD Spectrophotometer running Spectromanager in quartz cells with path length of 1 cm. CD samples were measured at 10 µM DNA, 10 mM Tris-HCl (pH 7.5), and 10 µM of respective ligand. Samples were annealed from 95 °C to 20 °C at a rate of -1.0 °C min^{-1}. To determine the stability of the G-quadruplex, the temperature 10 µM DNA with or without 10 µM ligand was added to the buffer consisting of 10 mM Tris-HCl (pH 7.5) and 10 mM KCl. The melting analyses were performed using a JASCO J-1500 equipped with a temperature control system. Before measurement, samples were annealed by cooling from 90 to 4 °C at −1.0 °C min^{-1}. The annealed samples were transferred into cuvettes and then the CD signal at 288 nm was collected with increasing the temperature from 4 to 95 °C at 0.5 °C min^{-1}. To determine thermodynamic parameters, the CD melting curves were normalized and analysed by curve fitting based on the two-state model in Kaleida Graph (Synergy Software) as per previous literature[10].

2. Supplementary Figures and Tables

2.1. Figure S1 – ^{1}H NMR Spectra

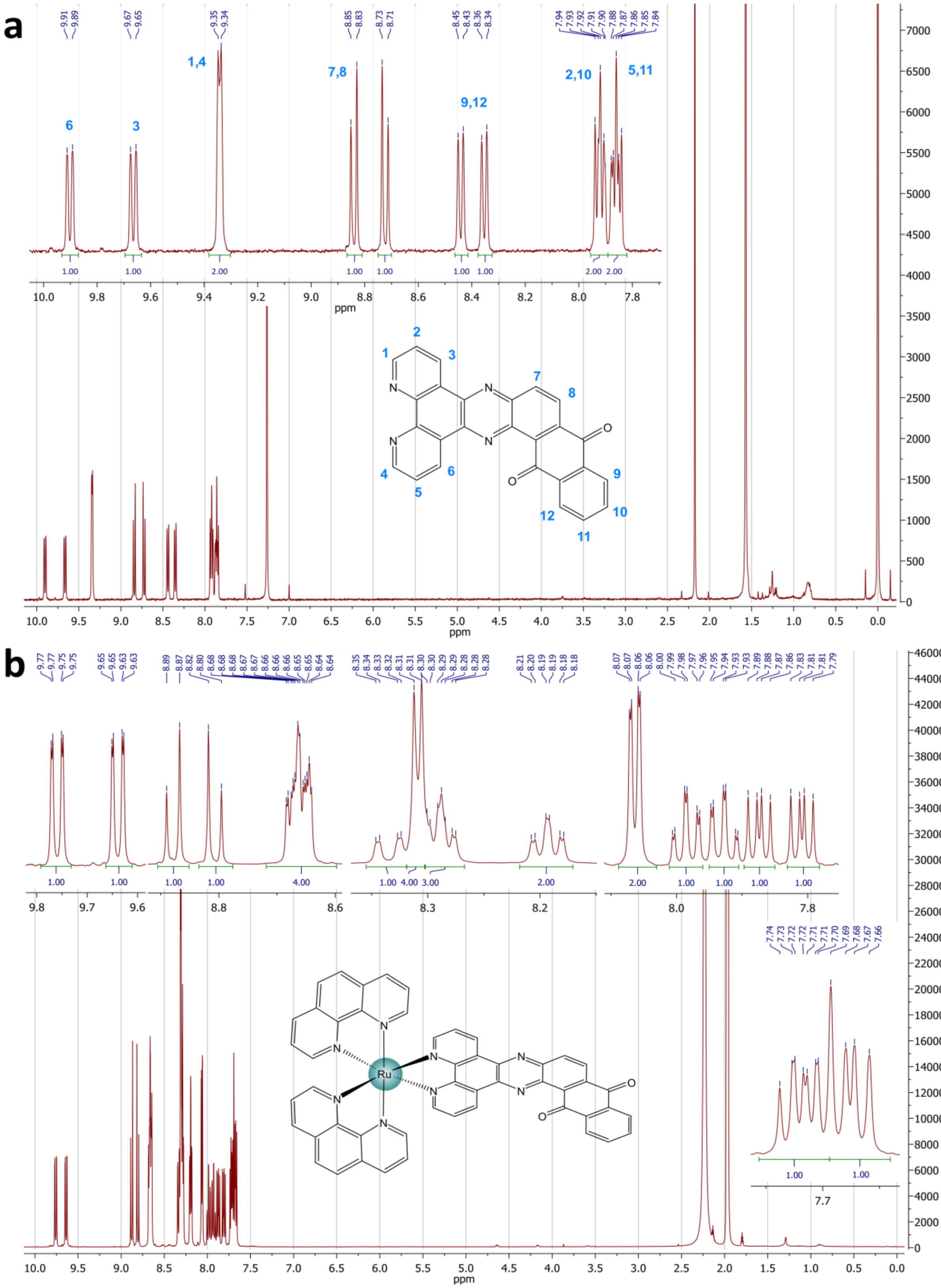

Figure S1 – *^{1}H NMR spectrum of (a) qdppz in $CDCl_3$, and (b) rac-[Ru(phen)$_2$(qdppz)]2PF$_6$ in ACN. Inset(s) shows magnified views of the aromatic regions. J-couplings and multiplet analysis can be found in section 1.3.*

2.2 Figure S2 – HPLC chromatograms (a and b) and CD spectra (c and d) of Λ/Δ-$[Ru(phen)_2(dppz)]^{2+}$ and Λ/Δ-$[Ru(phen)_2(qdppz)]^{2+}$, respectively

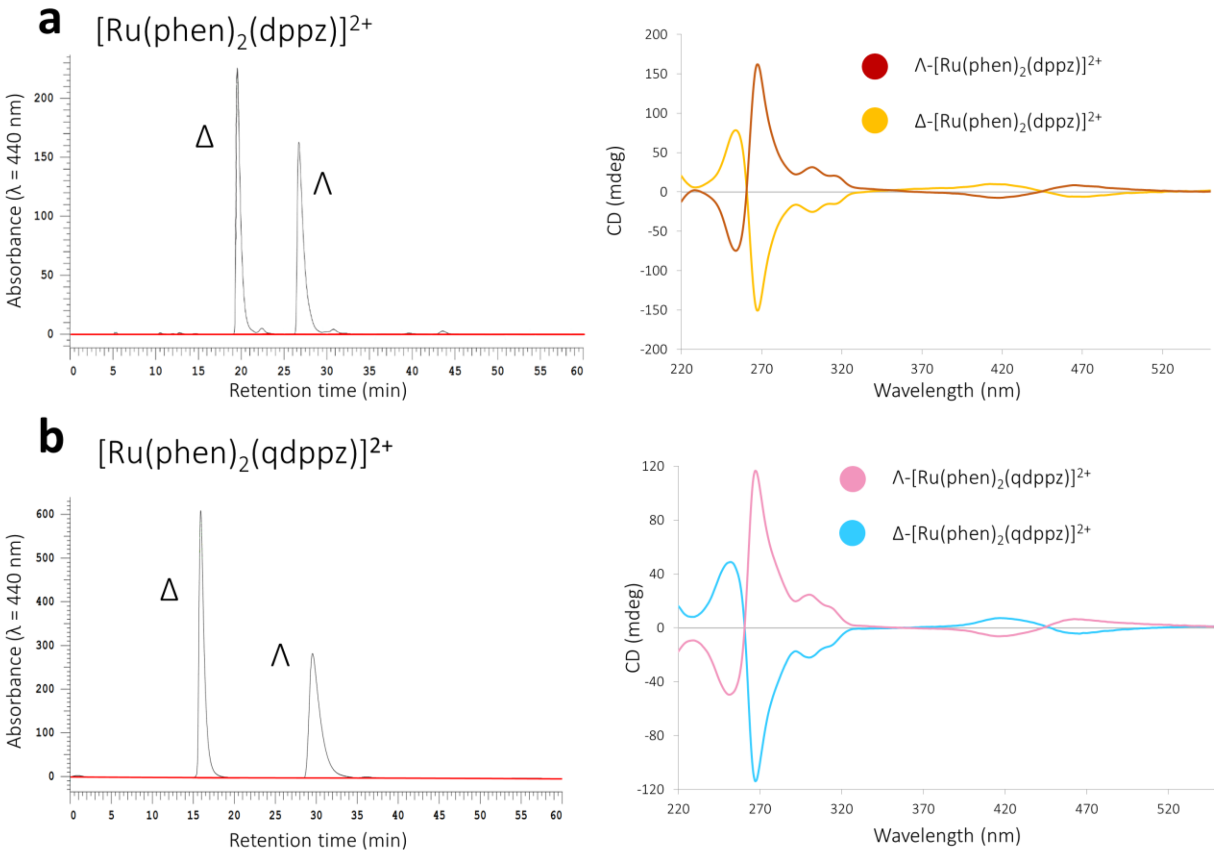

Figure S2 – *HPLC chromatograms and CD spectra of the enantiomers of (a)* $[Ru(phen)_2(dppz)]^{2+}$*, and (b)* $[Ru(phen)_2(qdppz)]^{2+}$ *following chiral separation. CD spectra were measured at 10 µM concentrations in ultrapure water.*

2.3 Table S1 - Data collection and processing parameters/refinement results for the small molecule dataset

Table S1 – *Collection and refinement parameters/statistics for the X-ray single crystal structure solution of Λ-[Ru(phen)$_2$(qdppz)]·Cl$_2$.*

Experimental Parameters	Λ-[Ru(phen)$_2$(qdppz)]·Cl$_2$
Empirical formula	$RuC_{50}N_8O_2H_{28}Cl_2$
Formula weight	944.78
Temperature (K)	100(2)
Crystal system	Triclinic
Space group	*P -1*
a (Å)	13.7372(2)
b (Å)	19.1244(2)
c (Å)	20.4059(2)
α (°)	69.529(1)
β (°)	88.509(1)
γ (°)	79.791(1)
Volume (Å)	4939.12(11)
Z	2
ρ_{calcd} (g cm^{-3})	1.271
μ (mm^{-1})	3.927
F_{000}	1912.0
Radiation wavelength (Å)	1.54184
R_{int}	0.048
Goodness of fit on F^2	1.039
**Data Refinement**	
Nº reflections	33180
Nº unique reflections	8909
Largest diff. peak and hole (eÅ^{-3})	1.73 and -0.91
Final R indices ($I > 2\sigma(I)$)	$R_1 = 0.0855$
R indices (all data)	$wR_2 = 0.2808$

2.4 Figure S3 – ORTEP model of the crystal structure of Λ-[Ru(phen)$_2$(qdppz)]$^{2+}$

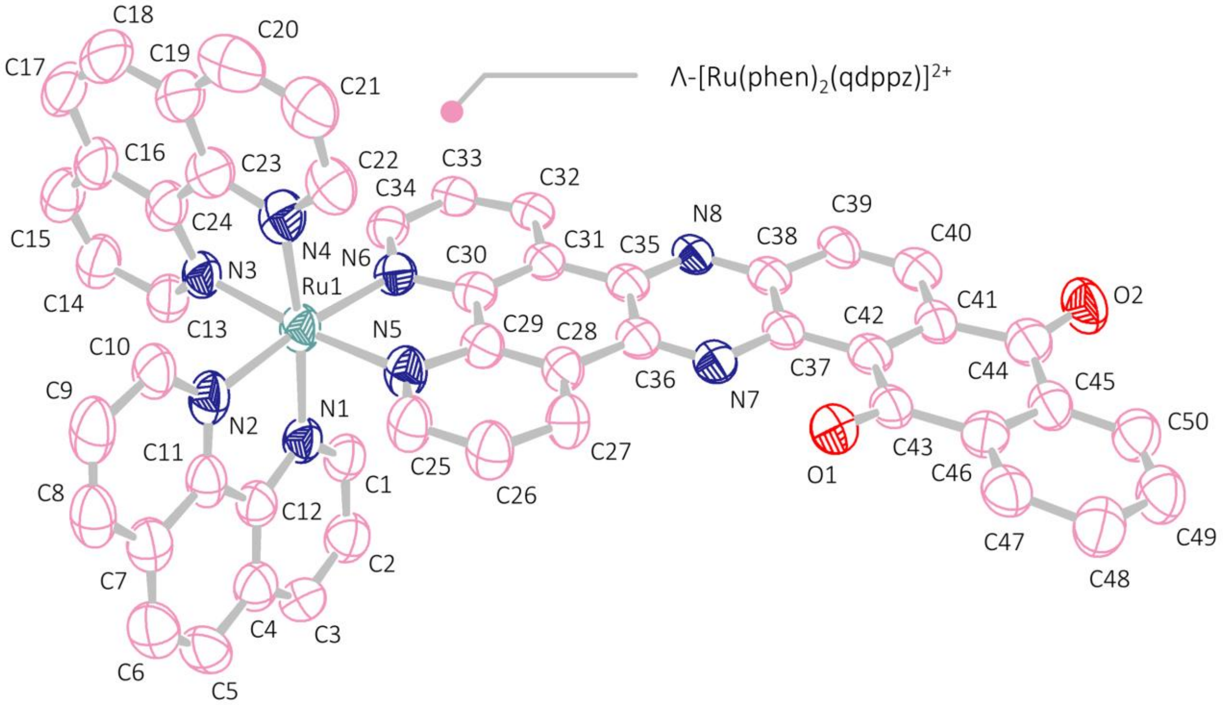

Figure S3 – *ORTEP representation and atom numbering of the final refined model of Λ-[Ru(phen)$_2$(qdppz)]·Cl$_2$. Thermal ellipsoids have been shown at the 50% probability.*

2.5 Table S2 – Selected bond, angles, and torsions from the small molecule dataset

Table S2 – *Selected bond lengths, angles and torsion angles from the small molecule dataset of Λ-[Ru(phen)$_2$(qdppz)]·Cl$_2$. Errors in parenthesis are quoted in the order of the smallest significant figure.*

Λ-[Ru(phen)$_2$(qdppz)]·Cl$_2$

**Selected Bond Lengths**					
Ru-N1	2.054(6)	Ru-N4	2.059(8)	C43-O1	1.232(9)
Ru-N2	2.076(6)	Ru-N5	2.045(6)	C44-O2	1.223(9)
Ru-N3	2.049(7)	Ru-N6	2.073(5)		
**Selected Bond Angles**					
N1-Ru-N2	79.9(3)	N2-Ru-N4	92.8(3)	N4-Ru-N5	95.7(3)
N1-Ru-N3	97.6(3)	N2-Ru-N5	95.1(2)	N4-Ru-N6	92.3(2)
N1-Ru-N4	172.4(2)	N2-Ru-N6	173.3(2)	N5-Ru-N6	80.1(2)
N1-Ru-N5	87.1(3)	N3-Ru-N4	79.9(3)	C42-C43-O1	122.7(7)
N1-Ru-N6	95.2(2)	N3-Ru-N5	174.7(3)	C41-C44-O2	120.6(9)
N2-Ru-N3	88.0(2)	N3-Ru-N6	97.1(2)		
**Selected Torsion Angles**					
N1-C12-C11-N2	-0.7(8)	N4-Ru-N3-C24	-9.0(4)	C37-C42-C43-O1	-5.3(7)
N1-Ru-N2-C11	7.9(5)	N5-C29-C30-N	-0.8(7)	C40-C41-C44-O2	8.2(7)
N2-Ru-N1-C12	-8.2(4)	N5-Ru-N6-C30	0.7(4)	C37-C42-C43-C46	175.3(6)
N3-C24-C23-N4	-1.1(7)	N6-Ru-N5-C29	-1.1(4)	C40-C41-C44-C45	-174.9(6)
N3-Ru-N4-C23	8.6(5)				

2.6 Figure S4 – Perspective views of the unit cells of the small molecule dataset

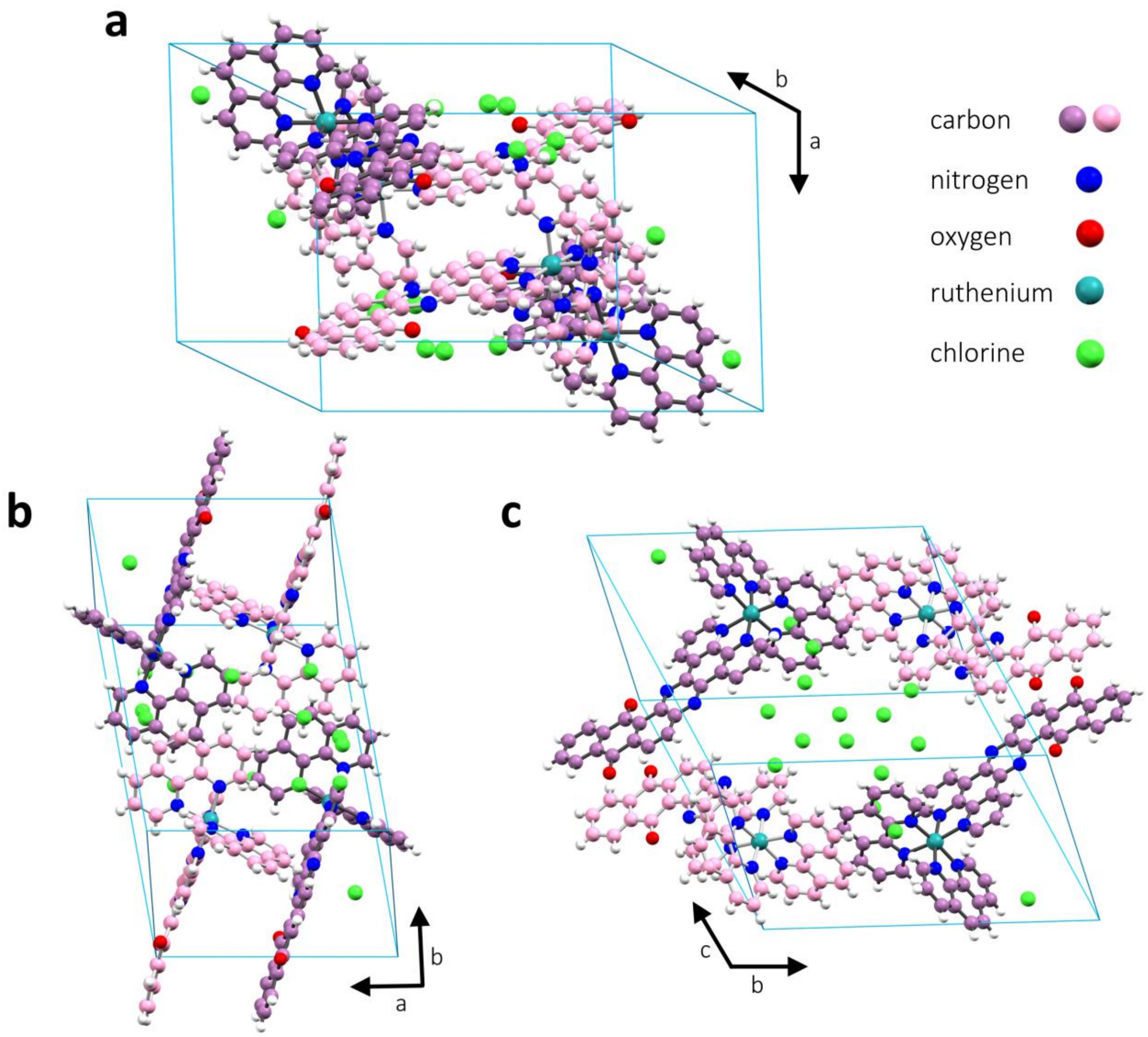

Figure S4 – *Perspective views of the unit cells from the crystal structure of Λ-[Ru(phen)$_2$(qdppz)]·Cl$_2$. Atom colours: carbon – light/dark pink, nitrogen – blue, ruthenium – teal, oxygen – red, and chlorine – green.*

2.7 Table S3 – Collection and processing statistics of 7OTB

Table S3 – *Collection and refinement parameters/statistics for the macromolecular X-ray single crystal structure solution of Λ-[Ru(phen)$_2$(qdppz)]·Cl$_2$ bound to (G$_3$T$_2$A)$_2$G$_3$T$_3$G$_3$ (PDB: 7OTB).*

	Λ-[Ru(phen)$_2$(qdppz)]$^{2+}$ with d((G$_3$T$_2$A)$_2$G$_3$T$_3$G$_3$)	
**Crystallisation Parameters**		
Crystal Morphology	Hexagonal Prismatic	
Growth Temperature (K)	277	
Crystal Size (µm)	30x30x90	
Growth Time	4 days	
**Data Collection**	*Anom. Dataset*	*High Res. Dataset*
Beamline	I03	I03
X-Ray Wavelength (Å)	1.7712	0.5603
Transmission (%)	2.97	30.01
Beamsize (µm)	20x20	20x20
Exposure Time (s)	0.05	0.05
№ Images/Oscillation (°)	3600/0.1	3600/0.10
Space Group	$P\,3_2\,21$	$P\,3_2\,21$
Cell Dimensions *a*, *b*, *c* (Å); α, β, γ (°)	29.77, 29.77, 114.27; 90, 90, 120	29.68, 29.68, 113.98; 90, 90, 120
**Data Processing**		
Resolution (Å)	28.57 - 1.81 (1.85 - 1.81)	25.07 - 1.44 (1.46 - 1.44)
R_{merge}	0.080 (0.377)	0.104 (1.416)
R_{meas}	0.085 (0.502)	0.107 (1.561)
R_{pim}	0.029 (0.328)	0.027 (0.640)
№ Observations	71,638 (247)	161,653 (2,694)
№ Unique Observations	5,344 (131)	11,038 (481)
I/σI	19.8 (1.7)	10.9 (0.3)
$CC_{1/2}$	0.998 (0.755)	0.999 (0.282)
Completeness (%)	90.3 (30.2)	98.0 (86.8)
Multiplicity	13.4 (1.9)	14.6 (5.6)
Mid-slope of anom normal probability	1.668	1.019
* Outer Shell Statistics Shown in Parentheses		
**Refinement**		
Phase Solution Method	SAD	
Resolution	21.29 (1.60)	
№ of Reflections	14,787	
R_{work}/R_{free}	0.1648/0.1810	
№ of Atoms		
DNA	845	
Metal Complex	89	
Water	113	
Average B Factors (Å^2)		
DNA	27.79	
Metal Complex	22.20	
Water	38.06	
rmsd		
Bond Lengths (Å)	0.021	
Bond Angles (°)	1.5	
PDB ID	7OTB	

2.8 Table S4 – Conformational analysis of 7OTB

Table S4 – *Conformational analysis, local base pair parameters, and base numbering for structure of* *Λ-[Ru(phen)$_2$(qdppz)]·Cl$_2$ bound to (G$_3$T$_2$A)$_2$G$_3$T$_3$G$_3$ (PDB: 7OTB).*

Local base-pair parameters

base pair	shear (Å)	stretch (Å)	stagger (Å)	buckle (°)	propeller (°)	opening (°)
$G_1 \cdot G_9$	-1.59	-3.43	0.85	-25.9	9.0	89.2
$G_9 \cdot G_{13}$	-2.05	-3.23	-0.83	13.3	5.6	89.8
$G_{13} \cdot G_{21}$	-1.65	-3.16	0.80	-24.3	5.9	94.1
$G_{21} \cdot G_1$	-1.70	-3.30	-0.63	9.6	4.7	91.4
anti-G_2·*syn*-G_8	1.51	3.37	-0.41	9.7	2.6	-88.4
syn-G_8·*anti*-G_{14}	1.28	3.80	0.28	-11.8	-8.4	-93.1
anti-G_{14}·*syn*-G_{20}	1.39	3.31	-0.40	11.8	2.2	-88.9
syn-G_{20}·*anti*-G_2	1.34	3.66	0.21	-11.5	-6.3	-92.1
syn-G_2·*anti*-G_8	-1.59	-3.43	0.85	-25.9	9.0	89.2
anti-G_8·*syn*-G_{14}	-1.31	-3.36	-0.25	16.6	7.3	91.2
syn-G_{14}·*anti*-G_{20}	-1.58	-3.54	0.54	-9.3	-7.9	90.9
anti-G_{20}·*syn*-G_2	-1.17	-3.61	-0.20	10.6	9.7	93.1
$G_3 \cdot G_7$	1.43	3.41	-0.65	20.6	-9.7	-89.2
$G_7 \cdot G_{15}$	1.41	3.42	0.64	-18.7	-4.7	-92.6
$G_{15} \cdot G_{19}$	1.31	3.51	-1.12	25.0	-11.1	-91.5
$G_{19} \cdot G_3$	1.40	3.37	0.51	-17.1	-0.1	-93.6
$T_5 \cdot T_{18}$	1.27	4.14	-2.20	61.8	8.6	113.5
$T_{10} \cdot A_{12}$	-0.84	3.71	0.24	-10.5	-13.3	-72.1
$A_{12} \cdot T_{11}$	0.11	-0.16	0.05	11.25	13.19	6.3

Local base step parameters

base step	shift (Å)	slide (Å)	rise (Å)	tilt (°)	roll (°)	twist (°)
G_1/*anti*-G_2	1.94	3.03	3.19	-77.5	147.3	-50.3
anti-G_2/G_3	-0.55	-0.08	3.25	7.2	0.4	24.6
G_1/*syn*-G_2	0.16	1.54	-3.37	2.8	-9.9	-25.1
syn-G_2/G_3	1.76	3.75	2.25	-79.9	150.6	-75.0
G_3/T_4	15.22	7.18	2.84	27.2	-36.6	139.8
T_4/T_5	-13.98	-1.86	1.02	-5.9	35.3	-73.1
T_5/A_6	3.21	1.14	9.32	-43.6	-32.9	67.9
A_6/G_7	-4.87	4.20	-9.46	54.3	53.1	-27.3
G_7/*anti*-G_8	0.24	1.55	-3.36	4.2	-5.3	-24.8
anti-G_8/G_9	1.13	4.13	0.64	-80.4	149.2	-136.6
G_7/*syn*-G_8	2.54	2.33	3.21	-76.9	148.8	-16.0
syn-G_8/G_9	-0.60	-0.23	2.99	7.0	8.2	22.4
G_9/T_{10}	5.56	4.41	-0.85	130.2	-101.7	79.7
T_{10}/T_{11}	0.66	-2.15	-3.37	-1.7	13.7	53.0
T_{11}/A_{12}	-0.33	-1.15	2.98	8.6	-4.8	20.3
A_{12}/G_{13}	2.27	6.17	-3.67	138.2	-79.5	61.1
G_{13}/*anti*-G_{14}	2.24	2.65	2.65	-74.6	152.6	-50.4
anti-G_{14}/G_{15}	-0.42	0.15	3.04	10.2	2.9	22.5
G_{13}/*syn*-G_{14}	-0.24	0.96	-3.29	0.8	-7.9	-25.8
syn-G_{14}/G_{15}	1.46	4.04	1.16	-78.2	150.0	-106.3
G_{15}/T_{16}	-1.09	-1.35	2.94	6.5	-1.6	24.9
T_{16}/T_{17}	-1.10	1.53	-7.10	26.6	84.5	49.2
T_{17}/T_{18}	3.95	-0.87	12.55	-65.3	-40.8	62.1
T_{18}/G_{19}	-4.98	2.36	-4.57	-14.9	44.9	-35.7
G_{19}/*anti*-G_{20}	0.30	1.79	-3.45	3.6	-4.2	-24.3
anti-G_{20}/G_{21}	0.99	4.25	0.36	-80.8	148.3	-131.7
G_{19}/*syn*-G_{20}	2.01	3.22	3.07	-79.2	151.7	-43.1
syn-G_{20}/G_{21}	-1.00	0.12	2.99	6.8	4.7	22.2

Local base-pair step parameters

base pair step	shift (Å)	slide (Å)	rise (Å)	tilt (°)	roll (°)	twist (°)
G_1*anti*-G_2:*syn*-G_8G_9	2.99	1.53	2.12	-84.6	157.1	6.5
anti-G_2G_3:G_7*syn*-G_8	0.24	-0.46	3.37	-0.7	-2.0	24.5
G_1*syn*-G_2:*anti*-G_8G_9	-0.18	0.45	-3.25	-0.9	-3.1	-23.6
syn-G_2G_3:G_7*anti*-G_8	-4.07	0.11	-0.07	82.0	-155.5	-172.3
G_7*syn*-G_8:*anti*-$G_{14}G_{15}$	0.85	0.53	-3.19	-2.9	-8.1	-23.7
syn-G_8G_9:G_{13}*anti*-G_{14}	-1.50	-3.20	-0.99	77.4	-150.9	136.4
G_7*anti*-G_8:*syn*-$G_{14}G_{15}$	1.50	3.41	1.29	-78.0	149.5	-132.3
anti-G_8G_9:G_{13}*syn*-G_{14}	-0.39	-0.69	3.12	2.0	8.5	24.3
G_{13}*anti*-G_{14}:*syn*-$G_{20}G_{21}$	-2.61	-2.28	-1.50	83.1	-159.1	30.8
anti-$G_{14}G_{15}$:G_{19}*syn*-G_{20}	0.44	-0.49	3.34	1.9	-2.4	23.3
G_{19}*syn*-G_{20}:*anti*-G_2G_3	0.91	0.72	-3.34	-2.6	-5.5	-24.4
syn-$G_{20}G_{21}$:G_1*anti*-G_2	-1.29	-3.50	-1.40	79.2	-147.8	139.3
G_{13}*syn*-G_{14}:*anti*-$G_{20}G_{21}$	-0.13	-0.18	-3.22	-0.9	-1.6	-23.7
syn-$G_{14}G_{15}$:G_{19}*anti*-G_{20}	3.71	0.58	1.28	-83.1	157.7	79.5
G_{19}*anti*-G_{20}:*syn*-G_2G_3	1.36	3.86	0.46	-79.8	151.3	-164.6
anti-$G_{20}G_{21}$:G_1*syn*-G_2	-1.01	-0.77	3.16	3.3	8.5	23.7
$T_{11}A_{12}$:$T_{11}A_{12}$	0.00	-1.17	2.99	0.0	-4.3	19.7

Local base step torsional angles

step	δ	ε	ζ	α	β	γ	δ	χ_1	NtC	CANA
G_1/*anti*-G_2	146.8	182.0	271.9	294.1	177.7	50.1	107.7	58.4	BBS1	SYN
anti-G_2/G_3	107.7	184.0	259.8	304.8	170.2	51.2	133.5	244.6	BB01	BBB
G_1/*syn*-G_2	146.8	183.0	272.5	296.3	178.6	46.6	108.2	58.4	NANT	NAN
syn-G_2/G_3	108.2	184.6	262.0	304.2	170.2	51.2	133.5	58.2	NANT	NAN
G_3/T_4	133.5	275.1	282.1	293.5	122.2	192.8	84.3	251.6	NANT	NAN
T_4/T_5	84.3	207.0	161.9	300.4	168.1	48.7	147.6	224.5	NANT	NAN
T_5/A_6	147.6	210.7	279.6	283.8	183.4	71.7	149.6	209.6	NANT	NAN
A_6/G_7	149.6	191.5	46.3	83.7	206.4	190.2	154.8	229.8	NANT	NAN
G_7/*syn*-G_8	154.8	167.5	281.2	286.3	189.5	53.5	107.6	70.5	NANT	NAN
syn-G_8/G_9	107.6	180.2	278.2	291.8	179.7	49.5	148.3	48.6	NANT	NAN
G_7/*anti*-G_8	154.8	181.4	268.1	302.2	169.5	53.1	117.5	70.5	BBS1	SYN
anti-G_8/G_9	117.5	186.2	261.5	290.7	179.7	49.5	148.3	245.4	BB00	BBB
G_9/T_{10}	148.3	178.2	279.7	150.3	99.4	195.1	72.1	263.6	NANT	NAN
T_{10}/T_{11}	72.1	209.9	61.8	149.5	159.0	49.8	118.6	53.0	NANT	NAN
T_{11}/A_{12}	118.6	205.4	298.2	251.4	73.3	170.0	150.4	220.8	NANT	NAN
A_{12}/G_{13}	150.4	242.7	279.4	138.4	141.1	176.7	161.4	202.2	NANT	NAN
G_{13}/*anti*-G_{14}	161.4	186.5	270.7	285.4	168.4	53.4	88.8	71.9	NANT	NAN
anti-G_{14}/G_{15}	88.8	178.9	270.4	300.9	165.7	67.7	130.4	229.0	AB01	A-B
G_{13}/*syn*-G_{14}	161.4	187.4	271.1	290.5	169.5	49.9	91.9	71.9	NANT	NAN
syn-G_{14}/G_{15}	91.9	180.4	267.8	300.0	165.7	67.7	130.4	61.6	NANT	NAN
G_{15}/T_{16}	130.4	173.8	263.2	294.9	186.0	58.7	133.8	238.5	BB00	BBB
T_{16}/T_{17}	133.8	192.9	257.5	202.4	162.9	42.3	143.1	241.6	NANT	NAN
T_{17}/T_{18}	143.1	203.9	292.2	293.1	232.3	60.4	133.8	211.2	NANT	NAN
T_{18}/G_{19}	133.8	258.3	60.0	87.2	196.9	189.1	149.7	233.1	NANT	NAN
G_{19}/*syn*-G_{20}	149.7	164.2	283.0	281.8	203.0	48.2	119.7	65.4	NANT	NAN
syn-G_{20}/G_{21}	119.7	196.5	252.4	308.0	158.5	48.0	133.2	60.6	NANT	NAN
G_{19}/*anti*-G_{20}	149.7	180.2	270.6	302.6	180.4	42.8	132.2	65.4	BBS1	SYN
anti-G_{20}/G_{21}	132.2	200.7	235.3	303.6	158.5	48.0	133.2	256.0	BB00	BBB

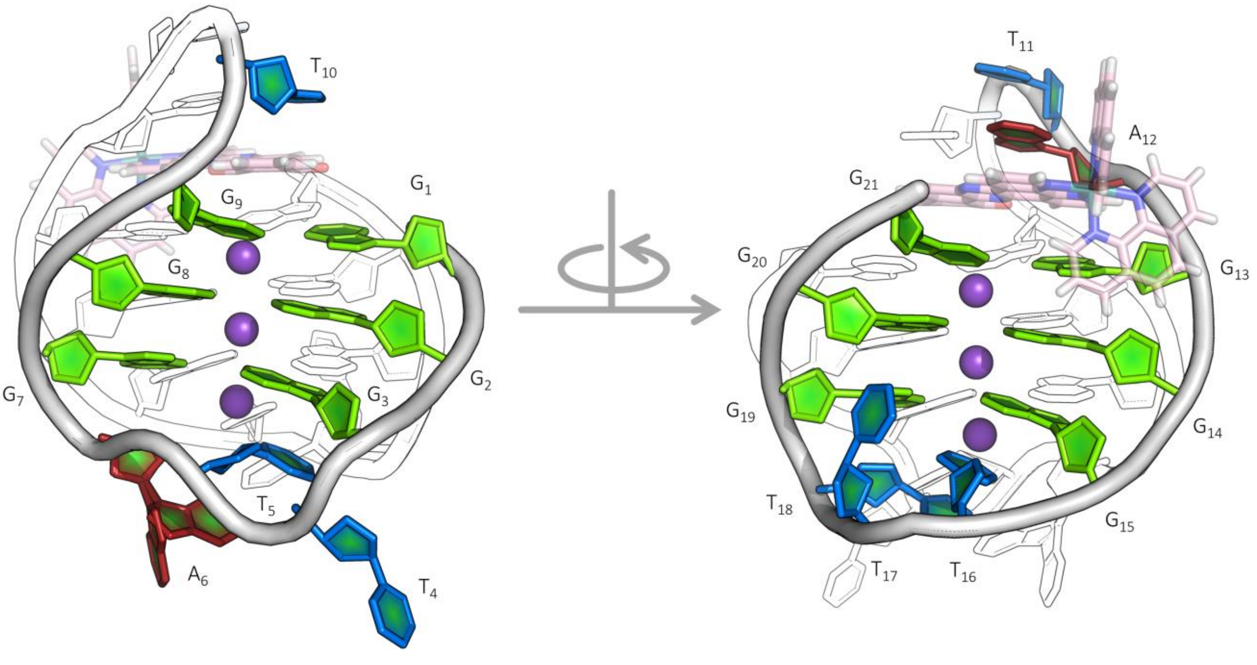

2.9 Figure S5 – Asymmetric unit of 7OTB

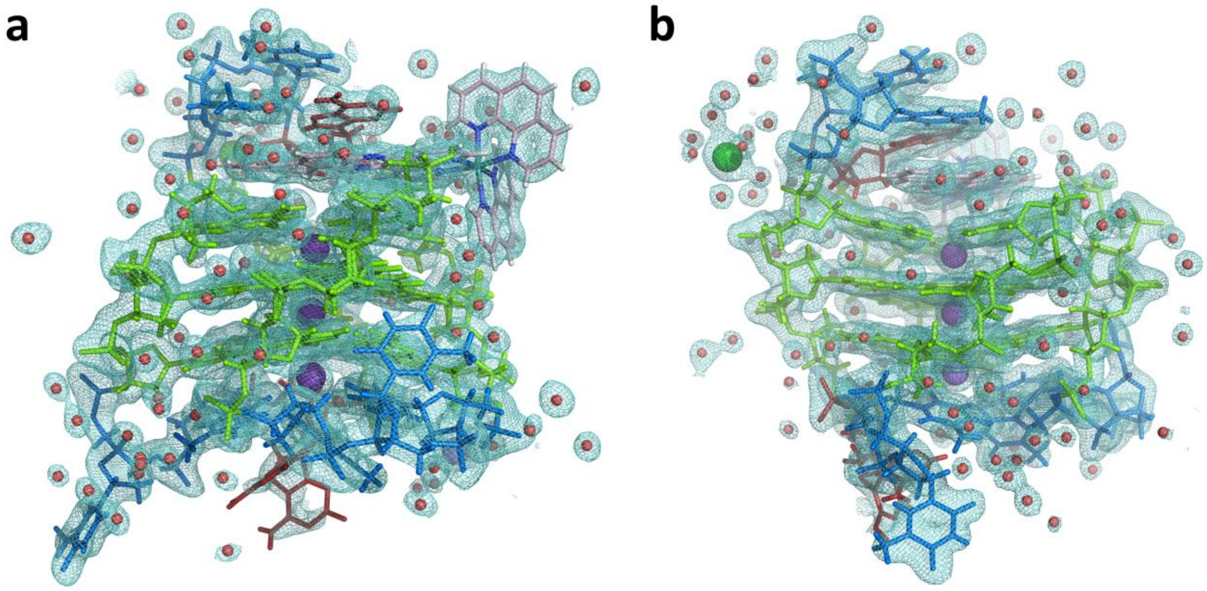

Figure S5 – *Two views, (a) and (b), of the asymmetric unit of 7OTB. 2Fo-Fc electron density map is shown and is contoured at a density of 0.39* e^{-} $Å^{-3}$. *Colour code for residues: guanine – green, thymine – blue, adenine – red; waters are shown as red spheres*.

2.10 Figure S6 – Ligand-adenine close contact

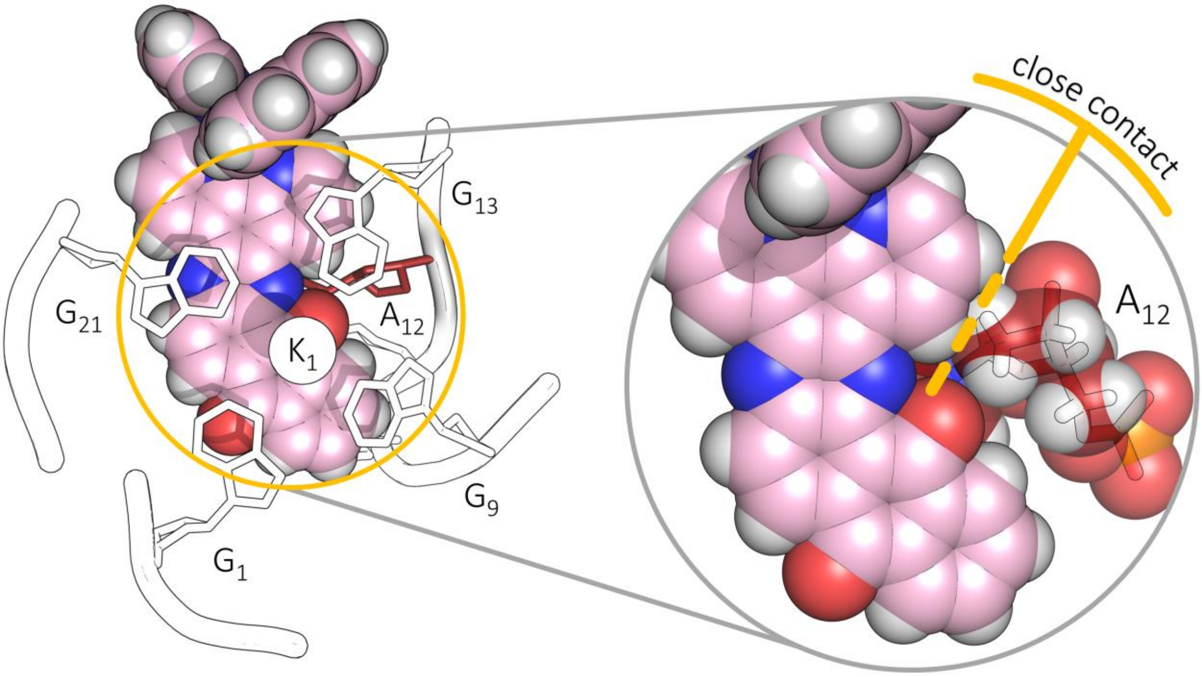

Figure S6 – *An adenine (A_{12}) from the central 'lateral' loop comes into close contact with the ruthenium complex. Specifically, the C2' of the ribose contacts the phenanthroline moiety of the qdppz ligand. This highlights a specific fit that would likely also be found with the wild telomeric sequence. Spherical representations are sized to the Van der Waals radius of the corresponding atoms.*

2.11 Figure S7 – Water/H-bonding network of 7OTB

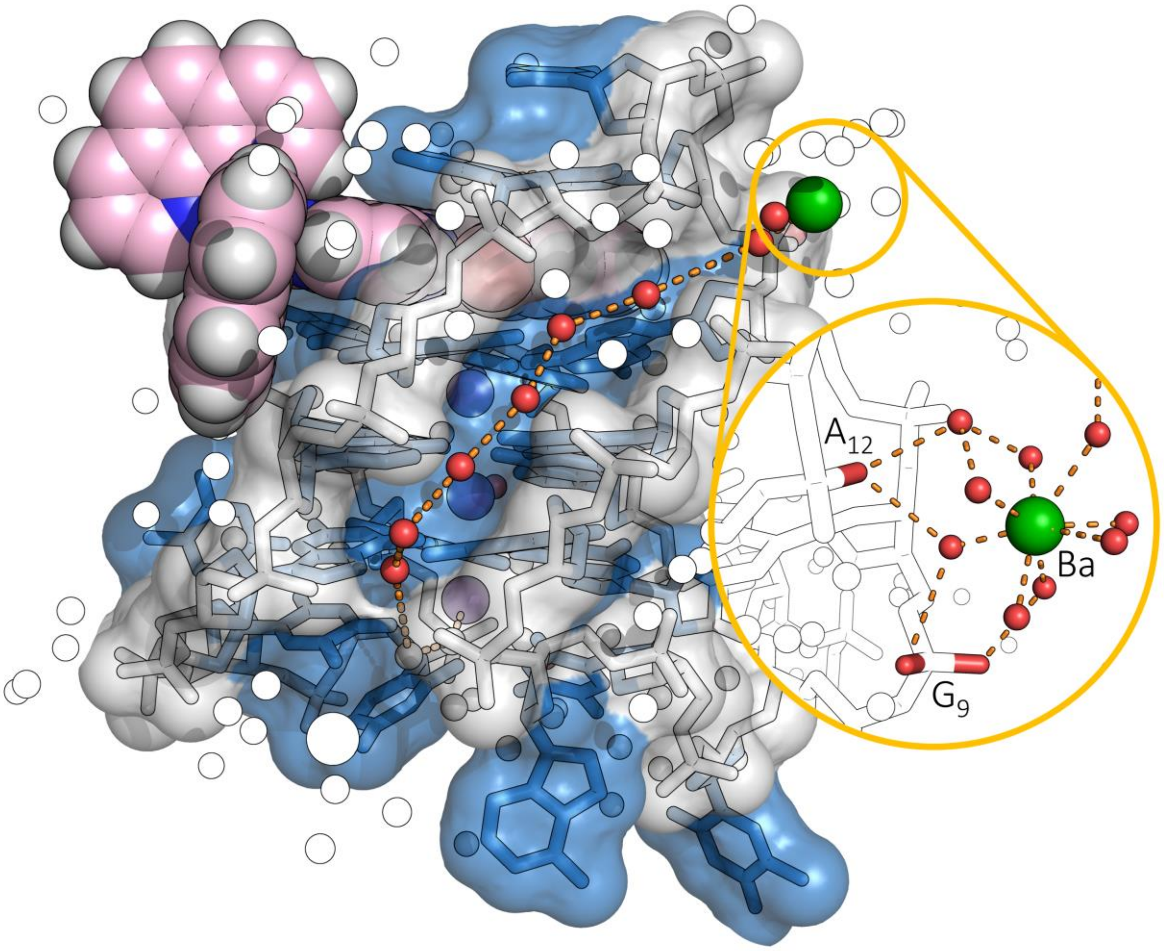

Figure S7 – *Graphic showing the calculated Van der Waals surface of the DNA, emphasizing the grooves and the water network found in the spine of the minor groove. Inset magnifies the coordination sphere around the barium to highlight the bridging nature of the cation between a terminal tetrad and the adjacent loop. In fact, the barium links multiple biological units via a fully occupied secondary hydration sphere, thus may be an important factor in the association of the crystal packing as seen in previously published DNA structures.*

2.12 Figure S8 – Central G-tetrad syn/anti order

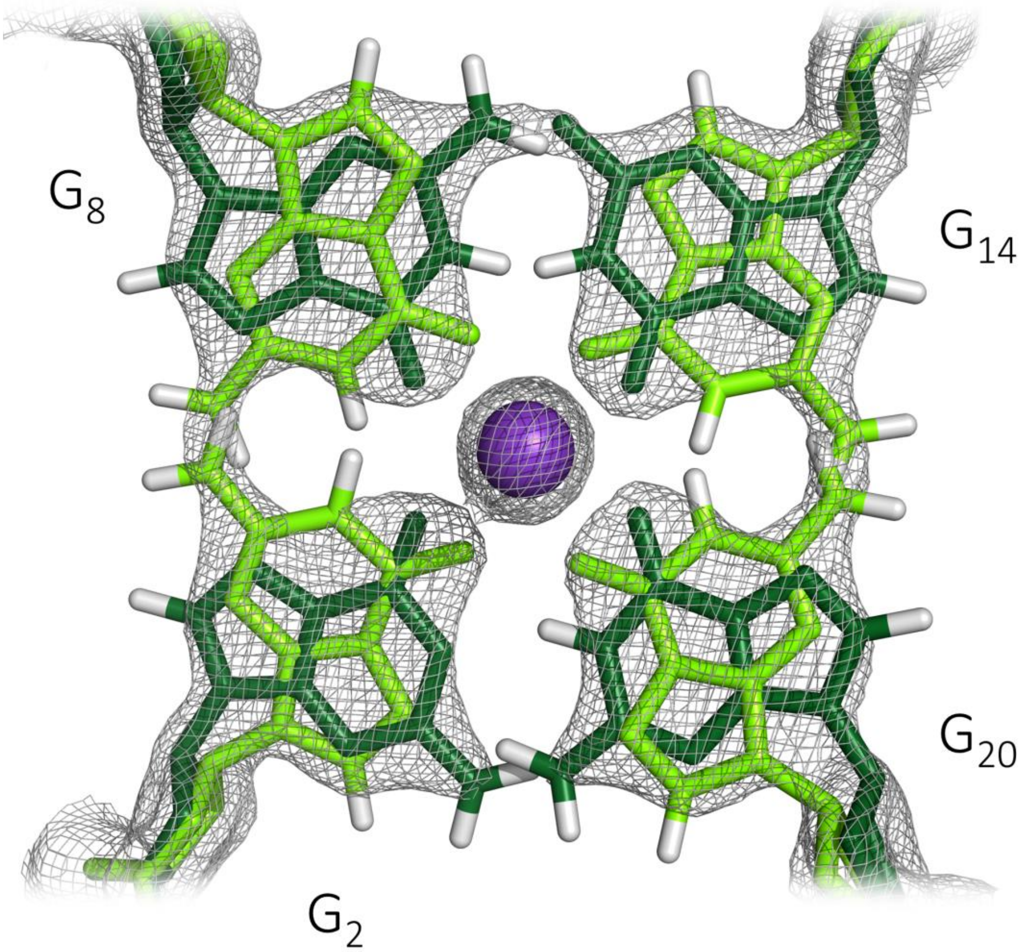

Figure S8 – *The central tetrad in the G-quadruplex is equally occupied between two conformations. In each case, the guanosine conformations alternate between syn (dark green) and anti (lime green) (i.e. G_8syn:G_{14}anti:G_{20}syn:G_2anti or G_8anti:G_{14}syn:G_{20}anti:G_2syn). The same observation can be seen in our previously published antiparallel structure; PDB code: 5LS8. 2Fo-Fc map is contoured at the 1σ level.*

2.13 Figure S9 – Superimposition of 7OTB and 6JKN

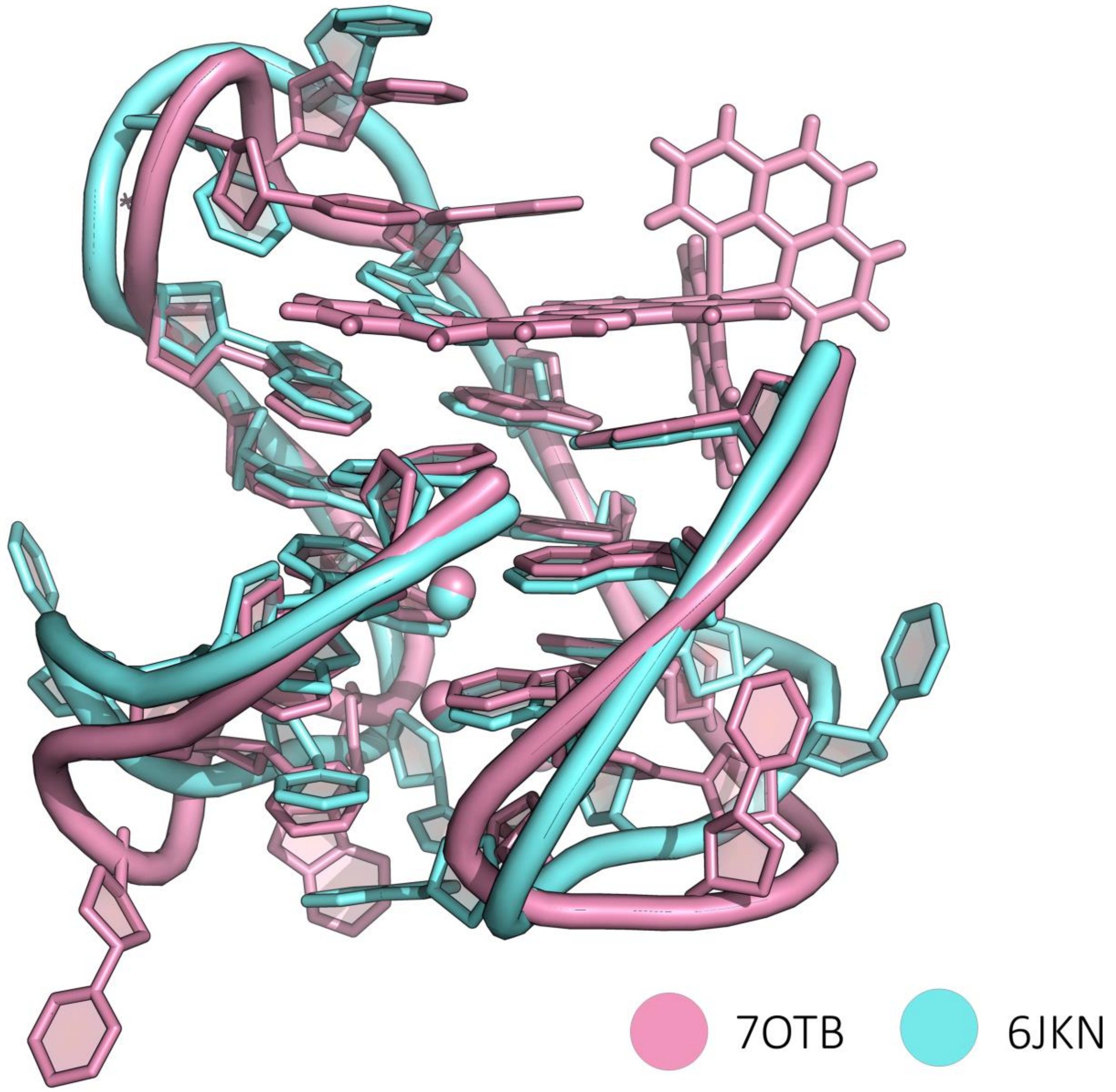

Figure S9 – *Superimposition of the herein described structure, 7OTB, with the previously published X-ray structure 6JKN. 6JKN contains a brominated guanine that promotes the antiparallel chair form. Note that both structures exhibit almost the exact same buckling within the G-tetrads.*

2.14 Figure S10 – Replication Analysis of $(T_2AG_3)_4$

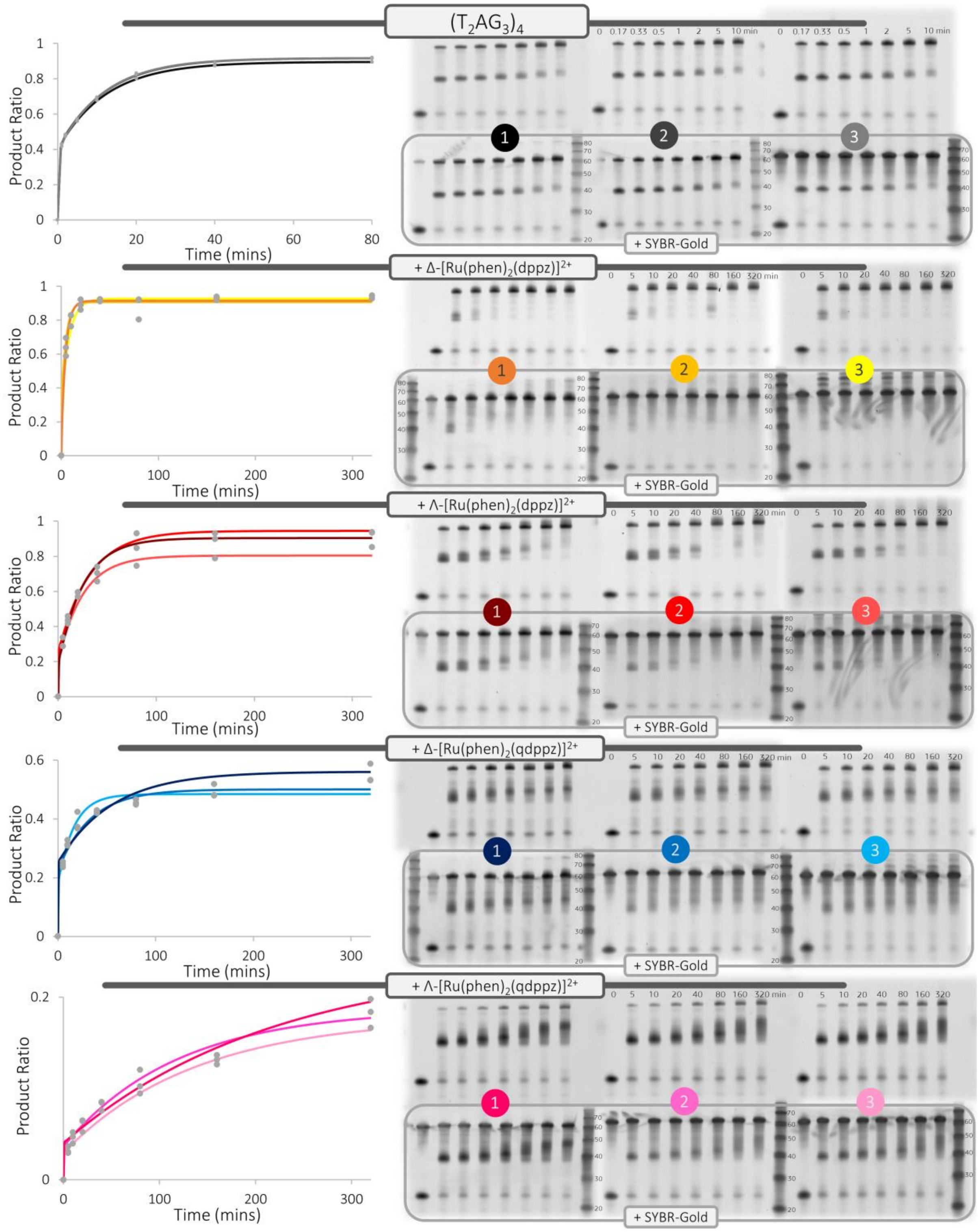

Figure S10 – *Denaturing PAGE electrograms and subsequent kinetic analyses of the replication of a d(T_2AG_3)$_4$ overhang, by the action of KF exo-, with and without the presence of the studied ruthenium complexes. Experiments were conducted at 1 μM DNA, 10 μM ruthenium complex, and 10 mM KCl. Each gel was then stained with SYBR-gold (inset) to reveal non-FAM labelled DNA (such as the DNA ladders). Full experimental details can be found in section 1.5.2.*

2.15 Figure S11 – Replication Analysis of $(G_3T_2A)_2G_3T_3G_3$

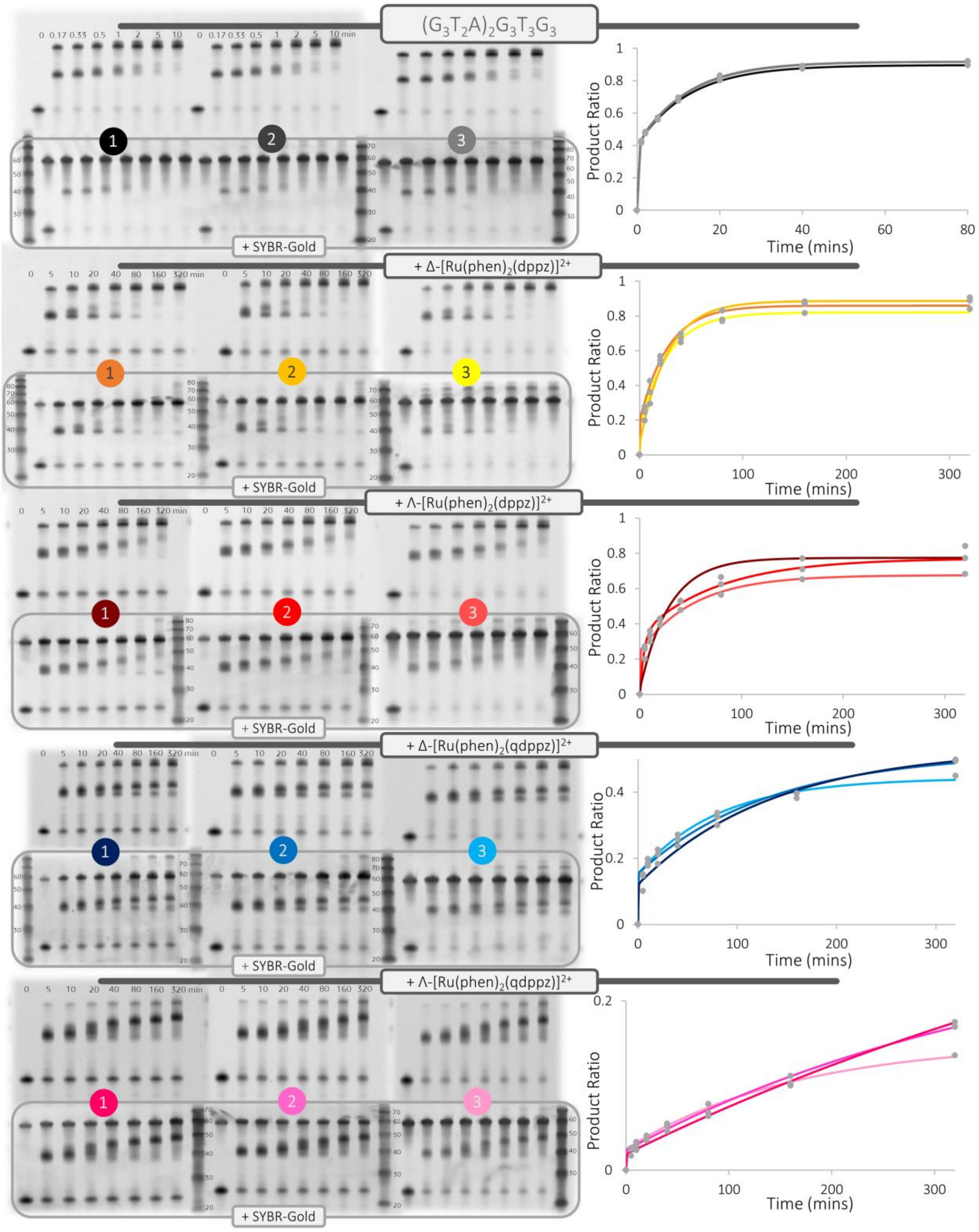

Figure S11 – *Denaturing PAGE electrograms and subsequent kinetic analyses of the replication of a d$(G_3T_2A)_2G_3T_3G_3$ overhang, by the action of KF exo-, with and without the presence of the studied ruthenium complexes. Experiments were conducted at 1 µM DNA, 10 µM ruthenium complex, and 10 mM KCl. Each gel was then stained with SYBR-gold (inset) to reveal non-FAM labelled DNA (such as the DNA ladders). Full experimental details can be found in section 1.5.2.*

2.16 Figure S12 – Replication Analysis of $(T_2AG_3)_4$ in LiCl

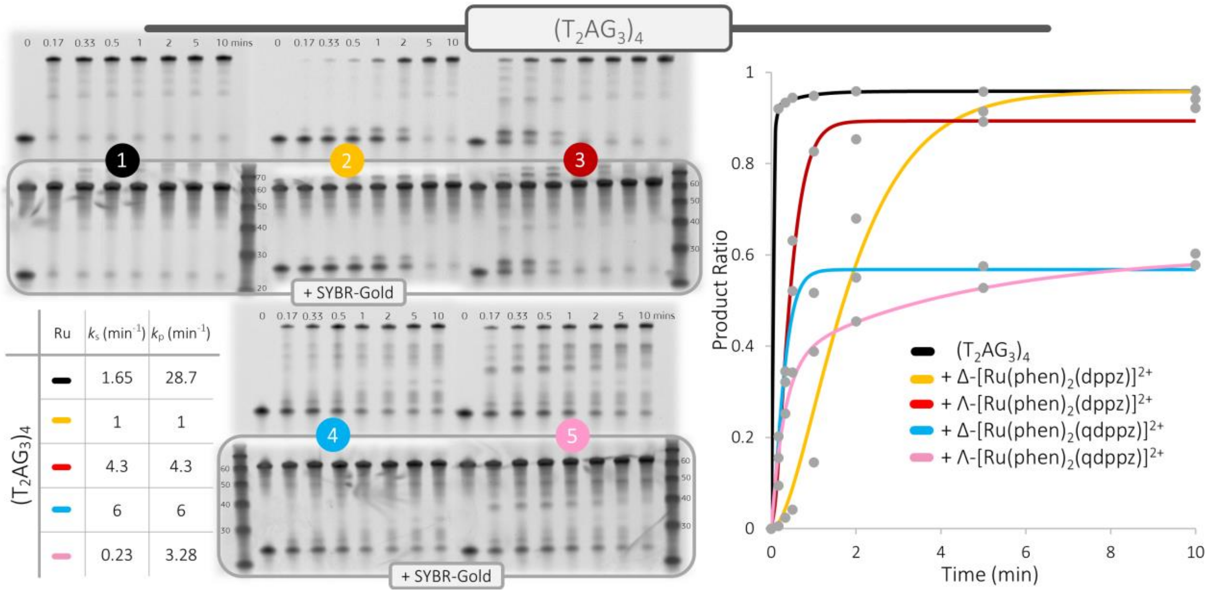

Figure S12 – *Denaturing PAGE electrograms and subsequent kinetic analyses of the replication of a d*$(T_2AG_3)_4$ *overhang, by the action of KF exo-, with and without the presence of the studied ruthenium complexes. Experiments were conducted at 1 µM DNA, 10 µM ruthenium complex, and 10 mM LiCl. Each gel was then stained with SYBR-gold (inset) to reveal non-FAM labelled DNA (such as the DNA ladders). Full experimental details can be found in section 1.5.2.*

2.17 Figure S13 – Replication Analysis of $(G_3T_2A)_2G_3T_3G_3$ in LiCl

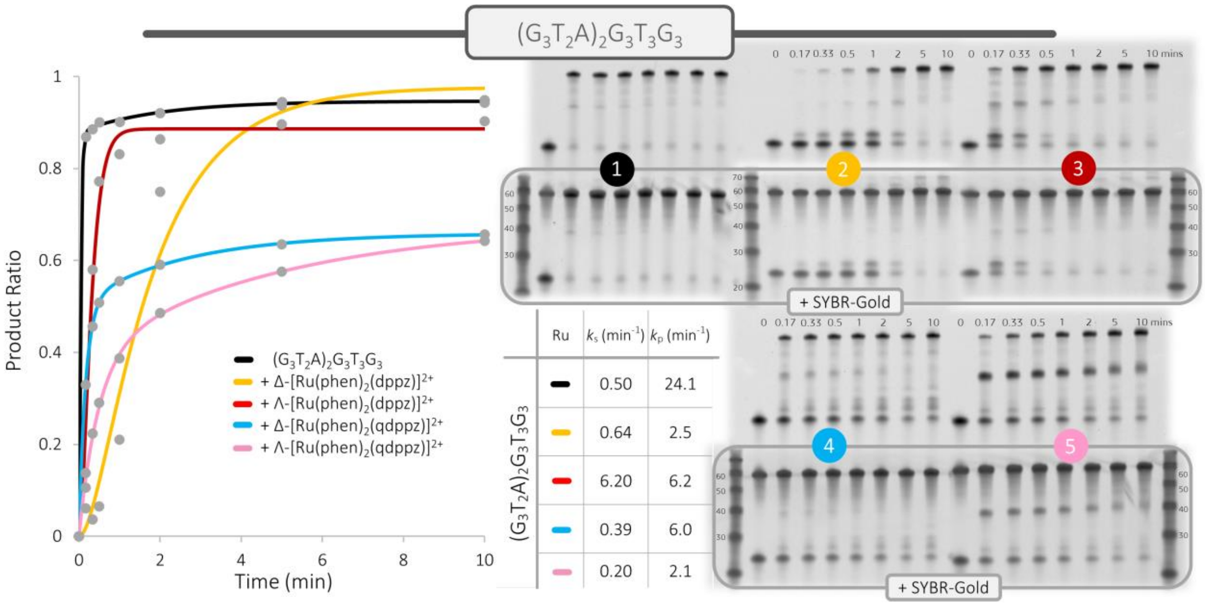

Figure S13 – *Denaturing PAGE electrograms and subsequent kinetic analyses of the replication of a d*$(G_3T_2A)_2G_3T_3G_3$ *overhang, by the action of KF exo-, with and without the presence of the studied ruthenium complexes. Experiments were conducted at 1 µM DNA, 10 µM ruthenium complex, and 10 mM LiCl. Each gel was then stained with SYBR-gold (inset) to reveal non-FAM labelled DNA (such as the DNA ladders). Full experimental details can be found in section 1.5.2.*

2.18 Table S5 – Kinetic analysis of the replication assays

Table S5 – *DNA melting temperatures (from CD experiments), free energy change, and rate constant analysis in triplicate for KF exo-replication studies in 10 mM KCl. Full experimental details can be found in section 1.5.2. and 1.6. Errors are quoted as a standard deviation(s) of the sample mean(s).*

	Additive	T_m (°C)		$-\Delta G^0_{37}$ (kcal mol^{-1})	k_s (min^{-1})				k_p (min^{-1})			
		x̄	ΔT_m	x̄	1	2	3	x̄	1	2	3	x̄
$(T_2AG_3)_4$	-	40.7 ±0.2	-	0.84 ±0.06	0.423	0.473	0.499	0.470 ±0.040	6.0	6.3	7.2	6.5 ±0.6
	+ Δ-[Ru(phen)$_2$(dppz)]$^{2+}$	46.5 ±0.8	5.8	2.24 ±0.60	0.130	0.220	0.220	0.190 ±0.050	9.5	9.6	9.9	9.7 ±0.2
	+ Λ-[Ru(phen)$_2$(dppz)]$^{2+}$	64.1 ±0.6	23.4	2.26 ±0.50	0.040	0.032	0.039	0.037 ±0.004	5.4	7.4	8.2	7.0 ±1.4
	+ Δ-[Ru(phen)$_2$(qdppz)]$^{2+}$	46.1 ±2.2	5.5	1.03 ±0.20	0.063	0.030	0.018	0.037 ±0.020	8.1	6.6	4.2	6.3 ±2.0
	+ Λ-[Ru(phen)$_2$(qdppz)]$^{2+}$	52.9 ±0.7	12.3	1.40 ±0.20	0.007	0.009	0.005	0.007 ±0.002	7.6	6.8	5.1	6.5 ±1.3
$(G_3T_2A)_2G_3T_3G_3$	-	51.6 ±0.1	-	3.09 ±0.46	0.081	0.082	0.085	0.083 ±0.002	3.8	2.7	3.1	3.2 ±0.6
	+ Δ-[Ru(phen)$_2$(dppz)]$^{2+}$	52.0 ±0.1	0.4	2.74 ±0.20	0.037	0.037	0.038	0.037 ±0.001	7.9	6.1	9.3	7.8 ±1.6
	+ Λ-[Ru(phen)$_2$(dppz)]$^{2+}$	63.1 ±1.3	11.5	2.78 ±0.14	0.019	0.013	0.035	0.022 ±0.010	5.5	0.3	11.0	5.6 ±5.4
	+ Δ-[Ru(phen)$_2$(qdppz)]$^{2+}$	57.2 ±0.2	5.6	2.89 ±0.50	0.012	0.008	0.007	0.009 ±0.003	4.5	4.0	3.2	3.9 ±0.7
	+ Λ-[Ru(phen)$_2$(qdppz)]$^{2+}$	59.2 ±0.4	7.6	3.65 ±0.43	0.006	0.003	0.001	0.003 ±0.003	7.4	5.7	2.3	5.1 ±2.6

2.19 Figure S14 – CD Thermal Melts of $(T_2AG_3)_4$ in the presence of ligands

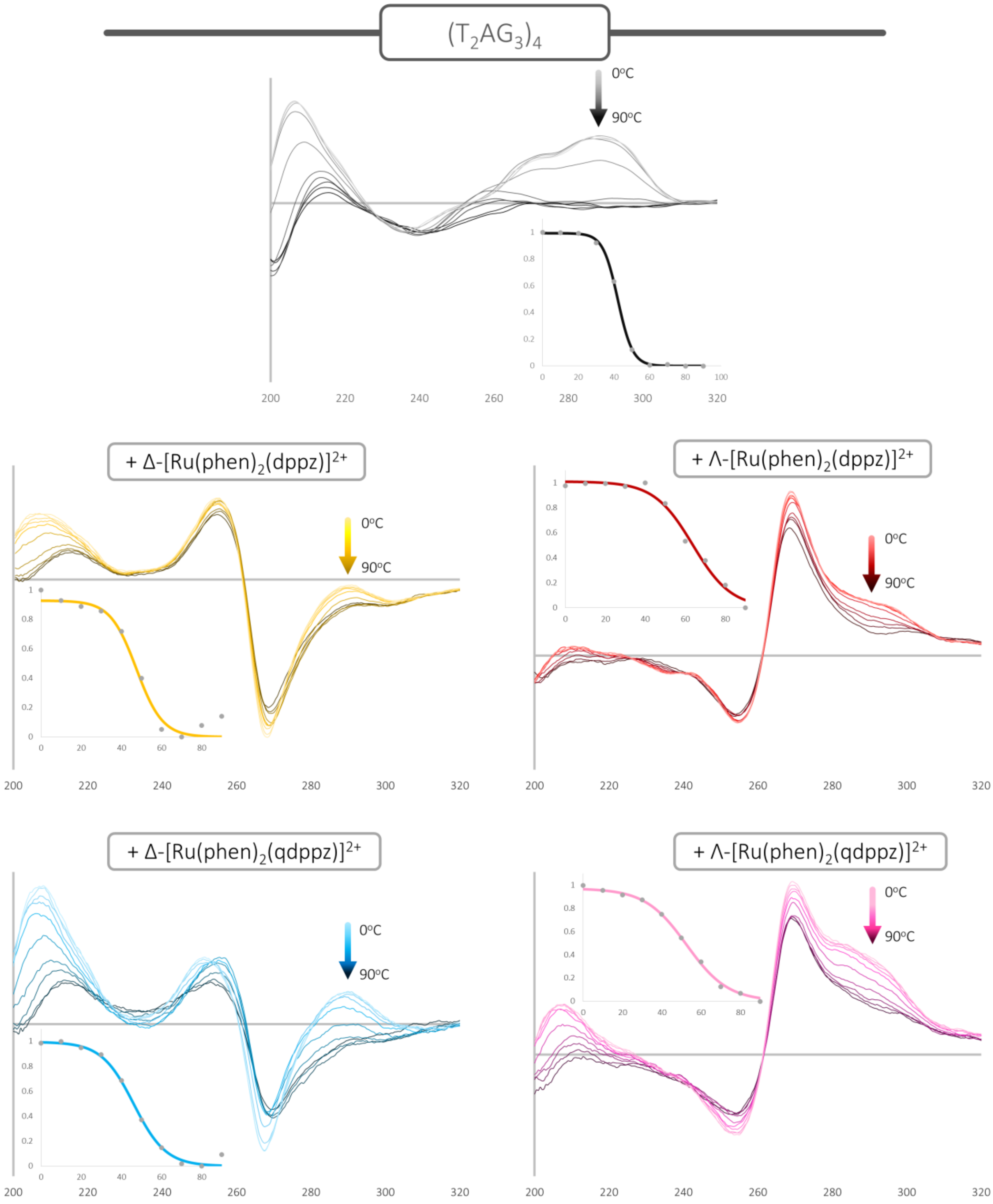

Figure S14 – *CD thermal melting profiles of d(T_2AG_3)$_4$ in the presence of each studied ruthenium enantiomer. Inset shows the isotherm of the hypochromicity at 290 nm (dots) along with the sitted sigmoid (line). Experiments were conducted at a DNA concentration of 10 µM and a ruthenium complex concentration of 10 µM in the presence of 10 mM KCl. Full experimental conditions can be found in section 1.6.*

2.20 Figure S15 – CD Thermal Melts of $(G_3T_2A)_2G_3T_3G_3$ in the presence of ligands

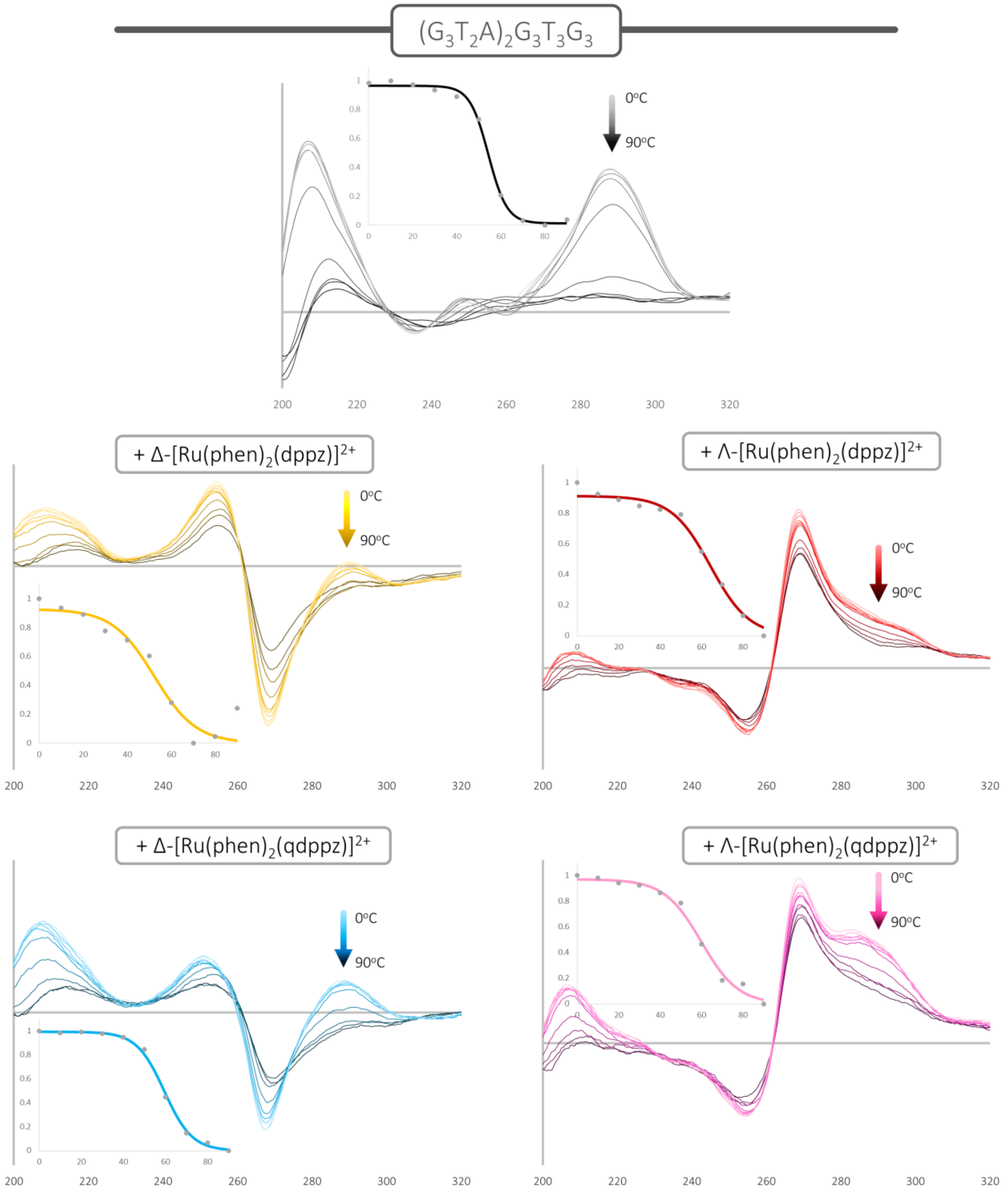

Figure S15 – *CD thermal melting profiles of d$(G_3T_2A)_2G_3T_3G_3$ in the presence of each studied ruthenium enantiomer. Inset shows the isotherm of the hypochromicity at 290 nm (dots) along with the sitted sigmoid (line). Experiments were conducted at a DNA concentration of 10 µM and a ruthenium complex concentration of 10 µM in the presence of 10 mM KCl. Full experimental conditions can be found in section 1.6.*

2.21 Figure S16 - CD melting profiles of $(T_2AG_3)_4$ and $(G_3T_2A)_2G_3T_3G_3$ in the presence of ruthenium complexes

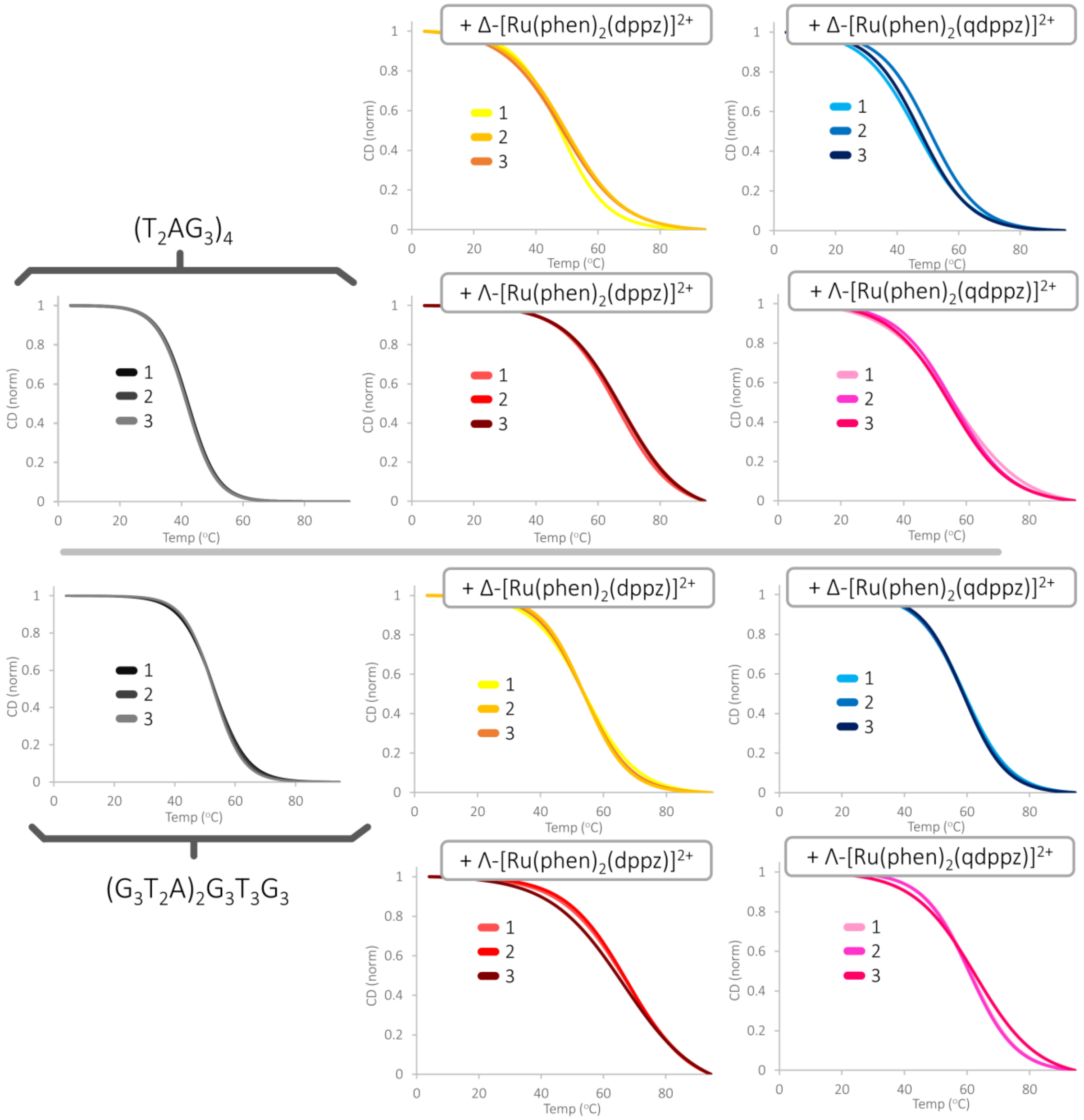

Figure S16 – *CD thermal melting profiles of d(T_2AG_3) and d(G_3T_2A)$_2G_3T_3G_3$ in the presence of each studied ruthenium enantiomer in triplicate. Each isotherm shows the hypochromic shift observed at 290 nm. Experiments were conducted at a DNA concentration of 10 µM and a complex concentration of 10 µM in the presence of 10 mM KCl. Full experimental conditions can be found in section 1.6.*

2.22 Table S6 Published antiparallel G-quadruplex structures

Table S6 – *Publicly available structures of ligand binding to antiparallel or hybrid G-quadruplexes. All structural data has been obtained using solution NMR techniques.*

Ligand	Sequence	Topology	PDB Code	Ref
Telomestatin Analogue	$T_2(G_3T_2)_3G_3A$	Hybrid 1 (3+1)	2MB3	11
ΔΔ-dinuclear Ru(II) Polypyridyl	$AG_3(T_2AG_3)_3$	Antiparallel Basket	2MCC	12
ΛΛ-dinuclear Ru(II) Polypyridyl	$AG_3(T_2AG_3)_3$	Antiparallel Basket	2MCO	12
Au-oxo6	$(T_2AG_3)_4TT$	Hybrid 2 (3+1)	5MVB	13
Pt(II) Tripod	$A_3(G_3T_2A)_3G_3A_2$	Hybrid 1 (3+1)	5Z80	14
Pt(II) Tripod	$A_3(G_3T_2A)_3G_3A_2$	Hybrid 1 (3+1)	5Z8F	14
Epiberberine	$(T_2AG_3)_4TT$	Hybrid 2 (3+1)	6CCW	15
Tripodal Ammonium Salt	$A_3(G_3T_2A)_3G_3A_2$	Hybrid 2 (3+1)	6KFI	16
Tripodal Ammonium Salt	$(T_2AG_3)_4TT$	Hybrid 2 (3+1)	6KFJ	16

3. References

1. Waterman, D. G. *et al.* The DIALS framework for integration software. *CCP4 Newslett. Protein Crystallogr* **49**, 13–15 (2013).

2. Evans, P. Scaling and assessment of data quality. *Acta. Crystallogr.* **D62**, 72–82 (2006).

3. Winter, G., Lobley, C. M. C. & Prince, S. M. Decision making in xia2. *Acta. Crystallogr.* **D69**, 1260–1273 (2013).

4. McCoy, A. J. *et al.* Phaser crystallographic software. *Journal of Applied Crystallography* **40**, 658–674 (2007).

5. Adams, P. D. *et al.* {\it PHENIX}: a comprehensive Python-based system for macromolecular structure solution. *Acta Crystallographica Section D* **66**, 213–221 (2010).

6. Emsley, P., Lohkamp, B., Scott, W. G. & Cowtan, K. Features and development of Coot. *Acta. Crystallogr.* **D66**, 486–501 (2010).

7. Adams, P. D. *et al.* The Phenix software for automated determination of macromolecular structures. *Methods (San Diego, Calif.)* **55**, 94–106 (2011).

8. Černý, J. *et al.* Structural alphabets for conformational analysis of nucleic acids available at dnatco.datmos.org. *Acta Crystallographica Section D: Structural Biology* **76**, 805–813 (2020).

9. Li, S., Olson, W. K. & Lu, X. J. Web 3DNA 2.0 for the analysis, visualization, and modeling of 3D nucleic acid structures. *Nucleic Acids Research* **47**, W26–W34 (2019).

10. Takahashi, S. *et al.* Chemical Modulation of DNA Replication along G-Quadruplex Based on Topology-Dependent Ligand Binding. *Journal of the American Chemical Society* **143**, 16458–16469 (2021).

11. Chung, W. J. *et al.* Solution Structure of an Intramolecular (3 + 1) Human Telomeric G-Quadruplex Bound to a Telomestatin Derivative. *J. Am. Chem. Soc.* **135**, 13495–13501 (2013).

12. Wilson, T., Costa, P. J., Félix, V., Williamson, M. P. & Thomas, J. A. Structural studies on dinuclear ruthenium(II) complexes that bind diastereoselectively to an antiparallel folded human telomere sequence. *J. Med. Chem.* **56**, 8674–8683 (2013).

13. Bazzicalupi, C. *et al.* Determinants for Tight and Selective Binding of a Medicinal Dicarbene Gold(I) Complex to a Telomeric DNA G-Quadruplex: A Joint ESI MS and XRD Investigation. *Angew. Chemie - Int. Ed.* **55**, 4256–4259 (2016).

14. Liu, W. *et al.* Solution structures of multiple G-quadruplex complexes induced by a platinum(II)-based tripod reveal dynamic binding. *Nat. Commun. 2018 91* **9**, 1–11 (2018).

15. Lin, C. *et al.* Molecular Recognition of the Hybrid-2 Human Telomeric G-Quadruplex by Epiberberine: Insights into Conversion of Telomeric G-Quadruplex Structures. *Angew. Chemie - Int. Ed.* **57**, 10888–10893 (2018).

16. Liu, L. Y. *et al.* Quantitative Detection of G-Quadruplex DNA in Live Cells Based on Photon Counts and Complex Structure Discrimination. *Angew. Chemie - Int. Ed.* **59**, 9719–9726 (2020).
